# Analysis of factors influencing surface settlement during shield construction of a double-line tunnel in a mudstone area

**DOI:** 10.1038/s41598-022-27206-7

**Published:** 2022-12-30

**Authors:** Chengwang Yuan, Min Zhang, Shengcai Ji, Jiwei Li, Lihaolin Jin

**Affiliations:** 1 College of Construction Engineering, Jilin University, Changchun, 130026 China; 2 China Railway 14th Bureau Group Co. Ltd., Jinan, 250101 China; 3 Beijing Urban Construction Explorationg & Surveying Design Institute Co. Ltd., Beijing, 100101 China

**Keywords:** Solid Earth sciences, Civil engineering

## Abstract

Urban rail transit is widely used in major cities worldwide due to its high efficiency, safety, and environmental friendliness. Shield construction has a fast excavation speed and a negligible impact on ground transportation; thus, it is the preferred construction method for urban rail transit tunnels. Mudstone is a widely distributed soft rock characterized by large deformation, low strength, and significant rheological differences in different areas. Mudstone causes problems in the design and construction of subways. This paper uses finite element analysis to establish a three-dimensional numerical model of a double-line tunnel in a weathered mudstone area and analyze the influence of the stratum, design, and construction parameters on surface settlement and deformation during asynchronous and simultaneous shield construction. The research results show that the lateral surface settlement curve obtained from the simulation is consistent with the measured data, demonstrating the reliability and feasibility of the three-dimensional numerical model. The surface settlement is affected by the deformation modulus, cohesion, and the angle of internal friction, and the deformation modulus has the most significant impact. The surface settlement decreases as the buried depth of the tunnel or the distance between the two center lines of the two tunnels increases. As the buried depth of the double-lane tunnel decreases or the distance between the two center lines of the two tunnels increases to a certain value, the lateral surface settlement curve exhibits two peaks. The surface settlement shows a decreasing trend with an increase in the thrust of the shield machine and an improvement in the grouting quality. However, excess grouting pressure causes surface uplift and a subsequent increase in surface subsidence.

## Introduction

Shield construction has a fast excavation speed and a relatively small impact on ground transportation. It has become the preferred construction method for urban rail transit tunnels and urban subway construction^1^. Surface settlement caused by shield construction and the resulting damage to adjacent buildings are important problems during subway construction, especially in certain types of strata^2^. Mudstone is a widely distributed soft rock prone to large deformation, has low strength, and exhibits significant rheological differences in different areas. Weathered mudstone disintegrates rapidly when it loses water and softens when it comes into contact with water. These characteristics result in challenges for shield tunneling in mudstone formations^3,4^.

The influence of different parameters on the surface settlement during shield construction of double-line tunnels in mudstone areas is unclear, complicating the prediction and control of surface settlement. Peck's equation is commonly used to analyze surface settlement during shield construction^5^. Suwansawat et al.^6^ analyzed surface settlement data of double-line shield construction in a clay layer in Bangkok and found that an empirical equation could be used to estimate surface settlement during the construction of a double-line tunnel or double-line superimposed tunnel. The tunneling pressure, grouting pressure, and other shield construction parameters significantly affected the settlement range and maximum settlement. Zheng^7^ used an empirical equation to analyze surface settlement caused by the tunnel construction of the Changchun Metro Line 1 through clay and mudstone layers. The result showed a good agreement between the measured data and theoretical calculations. The authors proposed a correction coefficient for the empirical equation suitable for the study area. However, empirical equations are typically only applicable to specific locations, require sufficient and accurate measurement data, and must be corrected using local measurements. Many factors affect surface settlement during shield construction of double-line tunnels, and it is highly variable. As a result, using empirical equations to predict surface settlement can be prone to errors^8^. Therefore, scholars have proposed analytical methods^9–11^, model tests^12–14^, numerical simulations^15–17^, and other methods to investigate surface settlement. Analytical methods assume that the formation is an ideal elastic or plastic body and focus on limited working conditions. The derivation process is complicated and is not universally applicable. Model tests utilize remolded soil to approximate certain conditions. However, this method is not suitable for mudstone, and the cost of in-situ tests is high. Numerical simulations use suitable soil constitutive relationships as ground parameters and separate the excavation process of the shield machine into several excavation steps, such as excavation, grouting, and segment assembly for the simulation. Numerical simulations have been used to analyze the influence of various factors on the surface settlement during shield tunnel construction^18^. Liu and Wang^19^ used finite element software to conduct 3D modeling of a section of the Guangzhou Metro tunnel in an area of weathered argillaceous siltstone. They conducted on-site monitoring and data analysis and found that surface settlement caused by shield tunneling was affected by multiple factors, such as the stratigraphic layers, groundwater level, buried depth of the tunnel, section characteristics, and construction factors. Wang^20^ used finite element analysis to investigate the impact of shield construction on surface settlement and the foundations of neighboring buildings. It was found that the degree of impact was related to the depth of the tunnel and the distance between the tunnel and the neighboring buildings. Guo et al.^21^ used numerical simulation to study surface settlement caused by tunnel excavation through clay and cobble layers. It was observed that the construction sequence affected the surface settlement and the shape of the surface settlement trough. Zhu et al.^22^ carried out a numerical simulation of tunnel construction in saturated loess in the Xi'an area. The authors analyzed the influence of the shield machine pressure, grouting pressure, groundwater level, and other factors on surface settlement and conducted sensitivity analysis. Li et al.^23^ focused on the subway tunnel project from Northeast Normal University Station to Gongnong Square Station of the Changchun Metro Line 1 and studied the site deformation characteristics caused by shield excavation of typical hard rock and soil composite strata using the discrete element analysis software FLAC3D. However, they did not consider the influence of the tunnel depth, grouting layer quality, deformation modulus, and other factors.

Although the above studies indicated that the surface settlement caused by the construction of double-line tunnels was related to many factors, few studies investigated tunnel construction in mudstone formations. Mudstone areas are widely distributed globally, and the scale of construction in mudstone areas is expanding. There is insufficient research on shield construction of double-line tunnels in mudstone areas, and further investigations are required to analyze the factors influencing surface settlement in mudstone areas. In this paper, numerical simulation is conducted, and a three-dimensional numerical model is established using as an example the shield construction of a tunnel between Bus Park Station to Jieda Road Station in Changchun City, Jilin Province. Three working conditions are analyzed and compared. In working condition 1, the excavation of one side is completed, and the excavation of the other side starts. Working condition 2 refers to the simultaneous excavation of the two tunnels in the same direction. Working condition 3 refers to excavating the left tunnel to a certain distance, followed by excavating the right tunnel (we call this asynchronous excavation). Working condition 1 and working condition 3 correspond to different stages of the shield construction process, and working condition 2 is a simplified method commonly used in numerical simulation to research the influence of the stratum, design, and construction parameters on the surface settlement and deformation of the double-line tunnel during shield construction. The results of this study provide guidance for the shield construction of double-line tunnels in mudstone formations.

## Engineering example

### Overview of the tunnel and geological conditions

**Figure 1:**
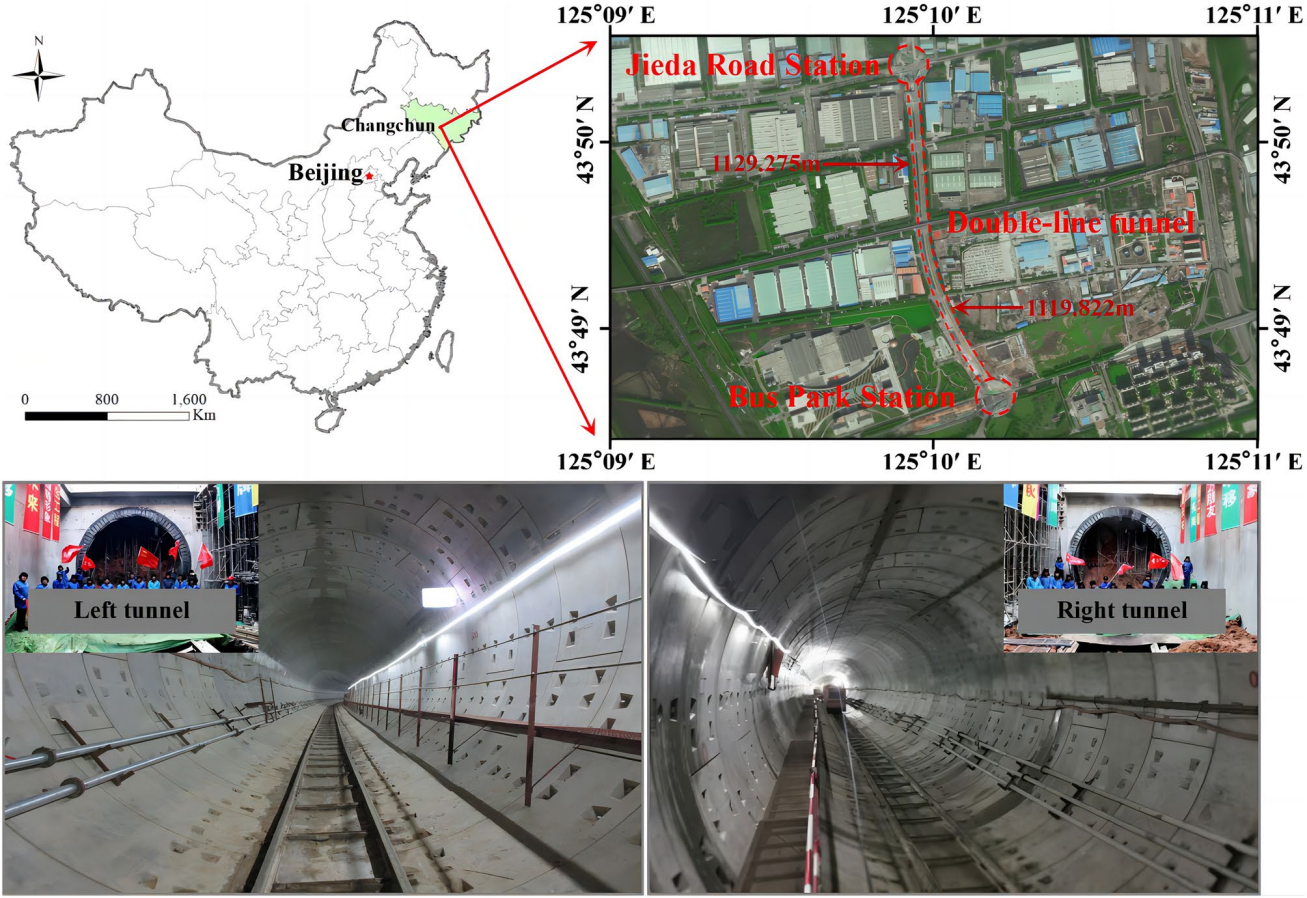
Engineering location and construction site diagram.

The rail transit of Changchun City, Jilin Province, China, has an operating mileage of 104.5 km; 135.4 km are currently under construction. The long-term plan is to build a 460 km rail transit network, which has great significance to the development of the city. This paper uses the shield-bored tunnel between Bus Park Station and Jieda Road Station in Changchun City, Jilin Province, China, as a case study (Fig. 1).

**Figure 2:**
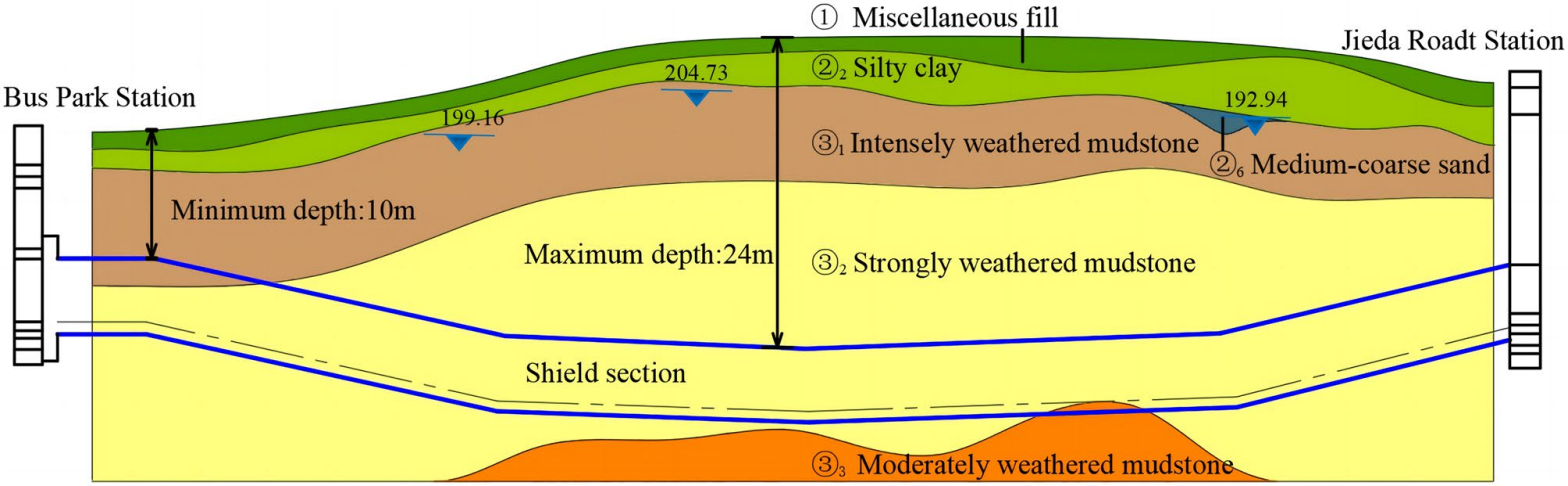
Schematic diagram of the geological section of the shield-bored tunnel between Bus Park Station to Jieda Road Station.

The length of the left tunnel of the shield section is 1129.275 m, and that of the right tunnel is 1119.822 m. The distance between the left and right tunnel centers is 14–30 m, and the roof of the tunnel is covered with soil with a thickness of 10–24 m. The overlying strata are ① miscellaneous fill, ②_2_ silty clay, ③_1_ intensely weathered mudstone, and ③_2_ strongly weathered mudstone. The tunnel passes through strata of ③_2_ strongly weathered mudstone, partially ③_1_ to intensely weathered mudstone, and ③_3_ moderately weathered mudstone. Weathered mudstone easily disintegrates after losing water and softens when it contacts water. If it is not supported in time, significant surface settlement or ground collapse can occur. Two layers of groundwater were detected in the study area. The first layer was porewater in silty clay with a water level ranging from 199.16 to 204.73 m, and the second layer was micro-confined water in sand with a water level of 192.94 m. A local aquifer was detected. The strongly weathered mudstone is an aquifuge layer; thus, there is no groundwater seepage. The schematic diagram of the geological section of the shield-bored tunnel between Bus Park Station and Jieda Road Station is shown in Fig. 2.

The relevant parameters were obtained from an indoor compression test, triaxial shear test, K0 consolidation test, and water content test. They are listed in Table 1.

**Table 1:** Physical and mechanical parameters of different strata.

Stratigraphic number	Thickness/m	Unit weight/(kN/m3)	Natural water content/%	Deformation modulus/MPa	Cohesion/kPa	Internal friction angle/°	Poisson ratio
①	1.60	18.0	17.5		5	8	0.20
②_2_	2.70	19.8	26.3	4.7	25	14	0.30
③_1_	7.55	20.3	21.3	10	35	15	0.26
③_2_	17.95	21.3	16.0	30	60	20	0.24
③_3_	10.20	22.7	12.0	100	120	35	0.21

Remarks: ① miscellaneous fill, ②_2_ silty clay, ③_1_ intensely weathered mudstone, ③_2_ strongly weathered mudstone, ③_3_ moderately weathered mudstone.

The tunnel was constructed by an earth pressure balance shield machine. After the left tunnel was excavated for 120 m, the shield machine began work on the right tunnel, i.e., we used an asynchronous excavation method.

### Construction monitoring scheme

**Figure 3:**
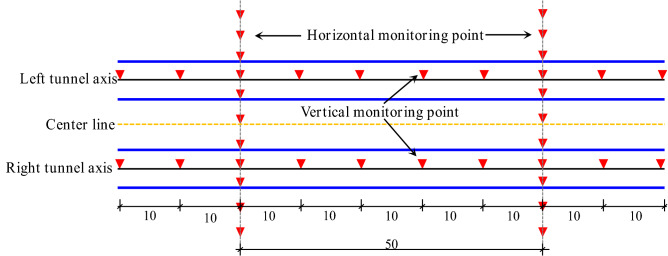
Plan view of the settlement monitoring points in the shield tunnel.

**Figure 4:**
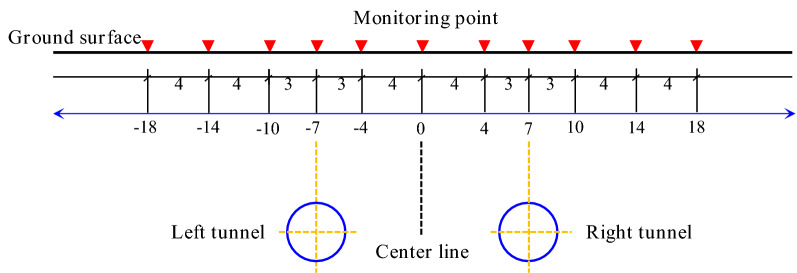
The layout of the settlement monitoring points in the cross-section of the shield section.

The vertical displacement of the ground surface was measured using geometric leveling and a Trimble DINI03 electronic level. The longitudinal settlement observation points were located above the tunnel axis, with a measuring point located every 10 m. The lateral settlement was measured at cross-sections every 50 m within the 100 m range of the shield starting and hoisting section, and monitoring cross-sections were located every 100 m in the remaining sections. The cross-section monitoring points were located at the centers of the left and right tunnels, with a spacing of 3~5 m. The layout of the settlement monitoring points of the shield tunnel is shown in Figs. 3 and 4.

**Figure 5:**
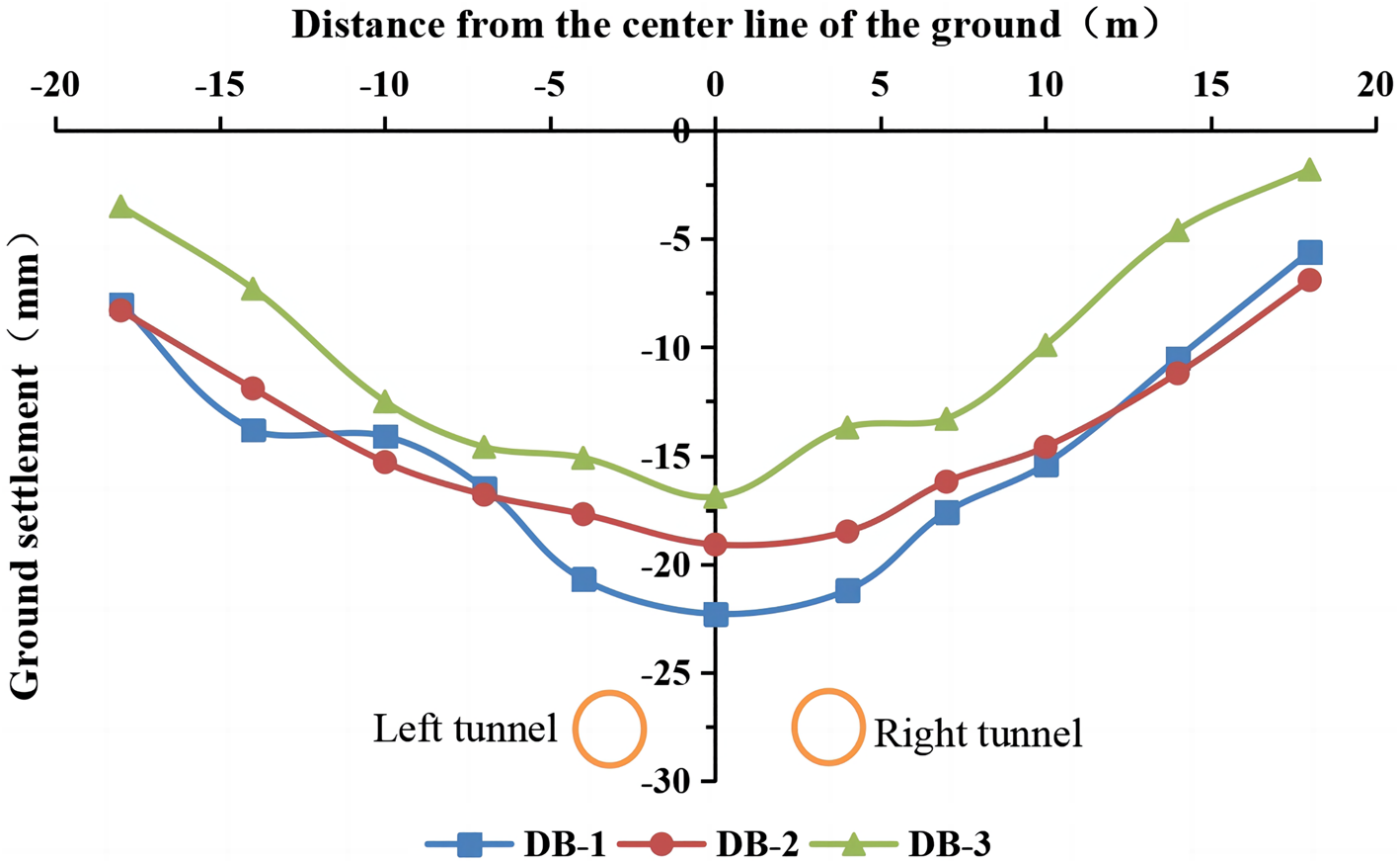
Measured surface settlement values after the excavation of the double-line tunnel.

We calculated the settlement amount, deformation rate, cumulative settlement, and other data in each construction stage using the elevation data at the monitoring points. After the excavation of the double tunnel was completed, the settlement of DB-1, DB-2, and DB-3 in the transverse monitoring sections was measured. The surface settlement curve is shown in Fig. 5.

The three surface settlement curves conform to Peck’s equation^5^. The tunnel’s burial depth is the shallowest in the DB-1 monitoring section, and the surface settlement is larger due to construction mode conversion. The burial depth is the deepest and the surface settlement is the smallest in the DB-3 monitoring section. The monitoring results provide information on the surrounding environment, the support structure, and the soil dynamics during construction, guiding the design and execution of subsequent construction projects.

## Three-dimensional numerical simulation

### Model description

#### Basic assumptions

Many factors affect surface settlement during the shield tunnel construction. It is not possible to consider all factors during the simulation because of model complexity, long calculation time, and potential errors. The following assumptions were made to simplify the calculation while achieving accurate results.

**Figure 6:**
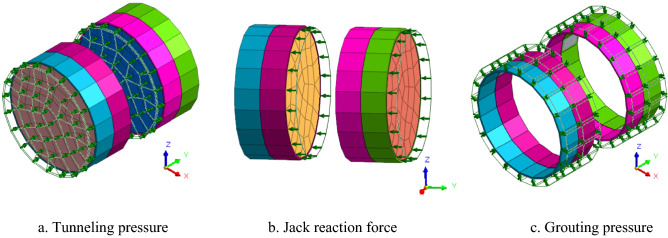
Diagram of the loading mode.

The layers are distributed horizontally, the average thickness of the stratum is used, and the Mohr–Coulomb model is used to model the material response^24^.The Mohr–Coulomb model is selected because its parameters are easy to obtain, and it is suitable for engineering applications. It can accurately simulate the stress–strain relationship of the strata. Its disadvantage is that it assumes elastoplastic deformation of the strata. The Mohr–Coulomb criterion is used to predict formation failure. An elastic model that does not consider material nonlinearity for structural materials such as segments and shield shells is chosen because the material has a large stiffness, and only elastic deformation occurs during construction. The groundwater level remains unchanged during the construction, and consolidation settlement caused by a change in the groundwater level due to construction is ignored. The tunneling pressure, jack reaction force, and grouting pressure during the shield construction process are simplified to apply uniform loads, and the average values of these parameters during the construction are used. The pressure on the excavation face, the jack’s thrust force on the segment, the cutter head torque, and the total thrust force during the excavation of the shield tunneling machine are the same in the simulation as in the actual conditions. The shield grouting is simulated by adjusting the grouting pressure and the material properties of the grouting layer. The loading mode is shown in Fig. 6.

#### Boundary conditions and three-dimensional model

**Figure 7:**
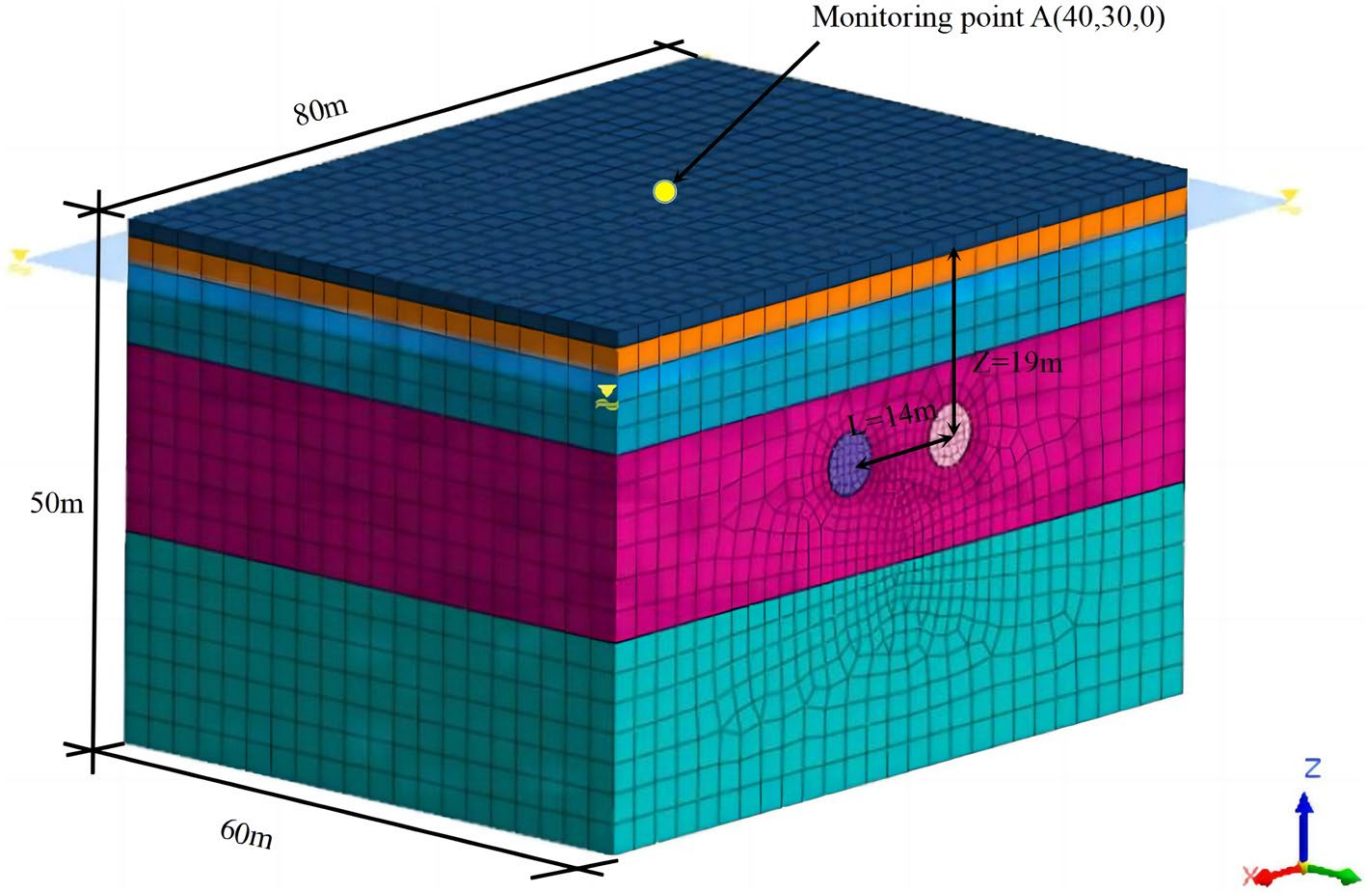
Three-dimensional model of tunnel excavation.

The shield tunnel construction was based on regional experience and on-site monitoring data. The impact range of shield construction is typically within 3 to 5 times the width of the tunnel, and the effect on the strata beyond this area is negligible. The tunnel in this case was a double-line circular tunnel with a single tunnel diameter of 6 m. The distance between the center lines of the two tunnels was 14 m, and the tunnel was buried at a depth of 19 m. The excavation was performed until reaching the 50th ring, which was an excavation length of 60 m. We considered the maximum value of the impact range in this simulation. The boundary of the model to the outside of the tunnel was 30 m. The simulation area for the first model establishment was 80 m × 60 m × 50 m. Displacement constraints were used at the boundary of the model, i.e., constraints in the X- and Y-directions were imposed on the side of the model to limit its horizontal movement. We applied constraints in the X-, Y-, and Z-directions at the bottom of the model. The top of the model represents the surface, and no constraints were imposed on the free surface. An initial stress field caused by the gravity load was applied to the model. The boundary conditions of the subsequent model were appropriately adjusted according to this principle. The three-dimensional model is shown in Fig. 7.

#### Model parameters

The strata parameters of the model were based on physical and mechanical tests of the strata. The physical and mechanical parameters of the strata and the parameters of the structural materials are listed in Tables 1 and 2.

**Table 2:** Parameters of the structural materials.

Name	Unit weight/(kN/m^3^)	Elastic modulus/MPa	Poisson ratio
Lining segment	25	30,000	0.20
Grouting layer	21	1.7	0.20
Shield shell	78	200,000	0.30

#### Numerical simulation

**Figure 8:**
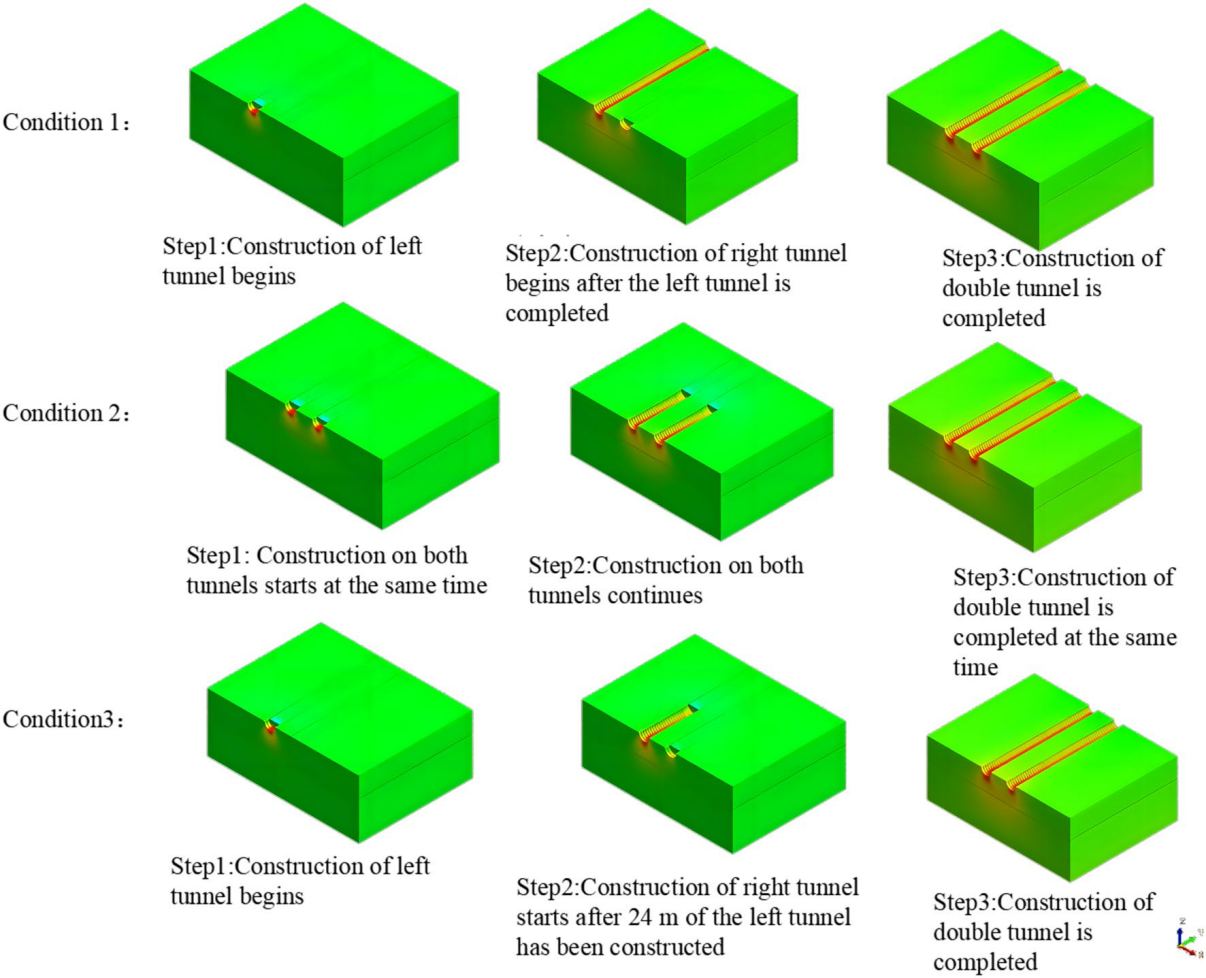
Construction sequence diagram of the three working conditions.

In the actual shield construction project, the left tunnel was excavated first. The right tunnel was excavated after the 100th ring was completed on the left line (120 m of excavation). Asynchronous excavation was used until the tunnel was completed. Researchers have typically simplified the construction process by assuming the following three working conditions. In working condition 1, the excavation is completed on one side of the tunnel, and the excavation of the other side begins. Working condition 2 refers to the simultaneous excavation of the two tunnels in the same direction. Working condition 3 refers to excavating the left tunnel to a certain distance, followed by excavating the right tunnel (we call this asynchronous excavation). The sequence of shield construction in the three working conditions is shown in Fig. 8.

### Simulation results and analysis

**Figure 9:**
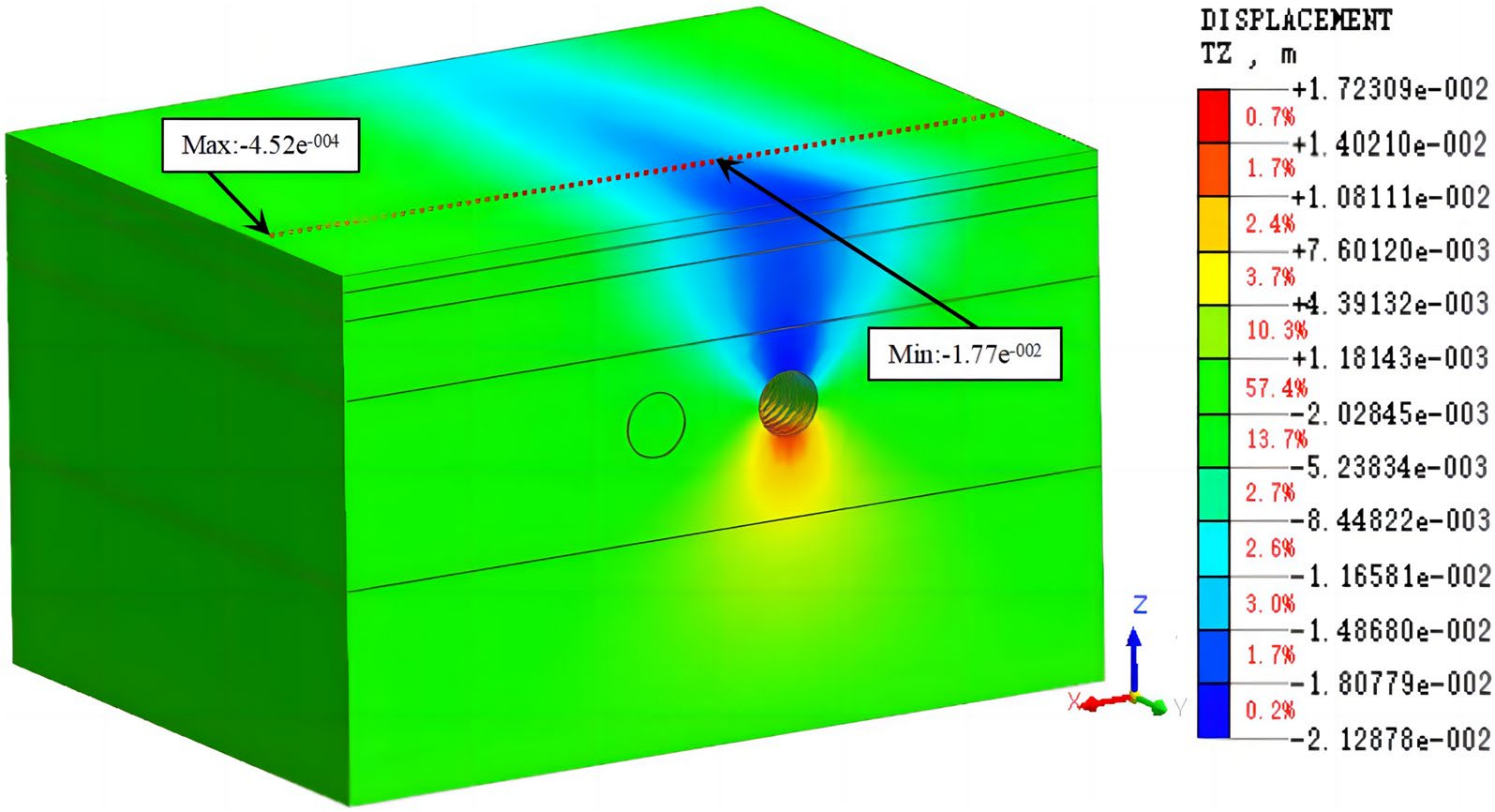
The cloud diagram of the vertical displacement for working condition 1 (excavation of the left tunnel).

**Figure 10:**
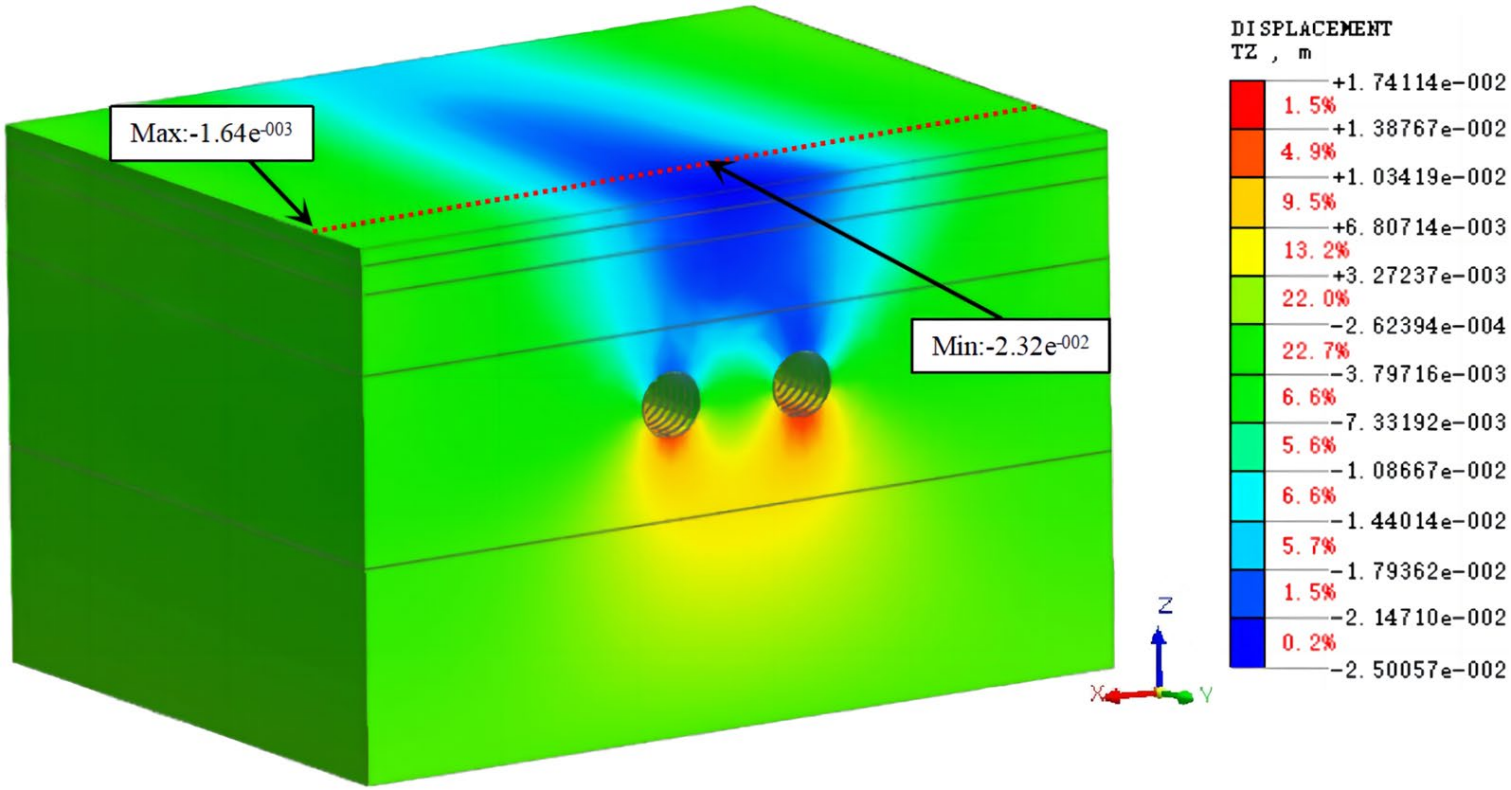
The cloud diagram of the vertical displacement for working condition 1 (excavation of both tunnels).

Working condition 1: This working condition simulated the construction process of the right tunnel after the completion of the 50th ring of the left tunnel, which is consistent with the excavation method of the first 100 rings in this example. The cloud diagrams of the vertical displacement of the tunnel after the excavation is completed are shown in Figs. 9 and 10.

**Figure 11:**
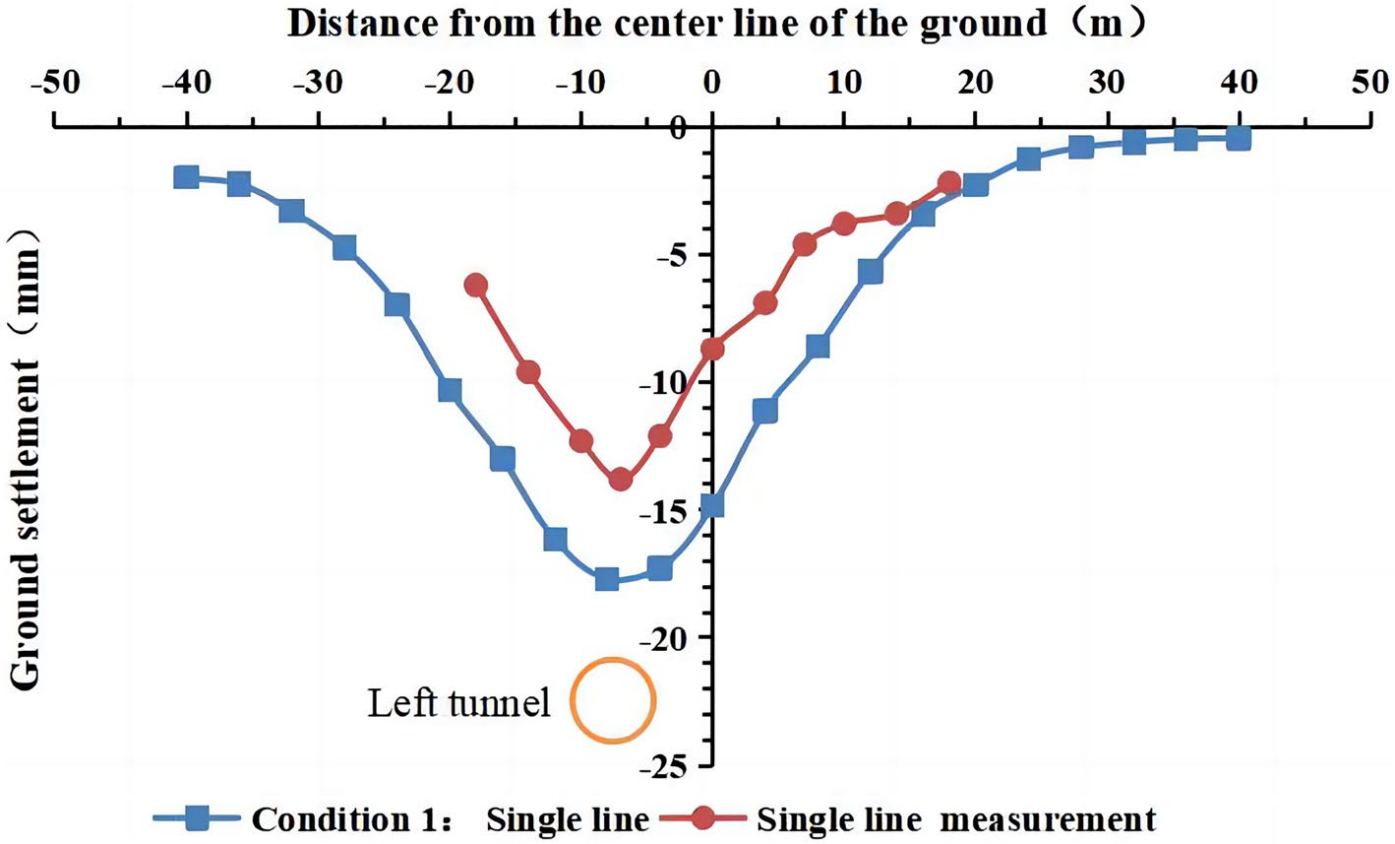
Comparison of the measured and simulated surface settlement after the excavation of the left tunnel.

**Figure 12:**
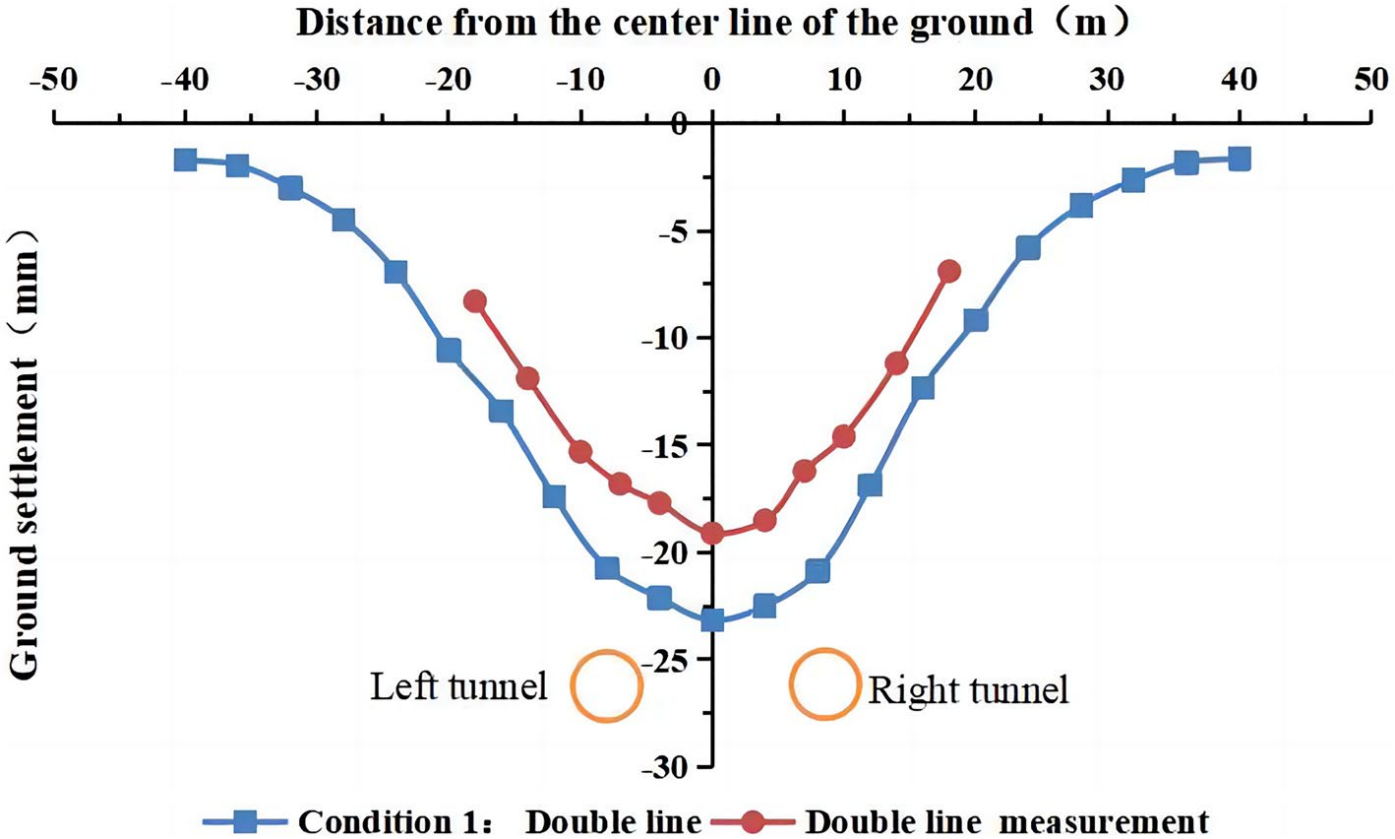
Comparison of the measured and simulated surface settlement after double-line excavation.

The comparison of the measured and simulated surface settlement values at the y = 50 m profile is shown in Figs. 11 and 12.

It is observed in Figs. 11 and 12 that the simulated and measured surface settlement values in the initial excavation section (the first 100 rings) for working condition 1 are similar, and the curves exhibit the same trend. After the completion of the single-line excavation of the left tunnel, the measured maximum surface settlement is 13.8 mm. This measurement point is located above the central axis of the tunnel. The curves in Figs. 9 and 10 are V-shaped. The maximum settlement value obtained from the simulation is 18.2 mm. After the excavation of the right line is completed, the measured maximum surface settlement is 19.1 mm. This measurement point is located above the midpoint of the line connecting the central axis of the left and right tunnels. The settlement value of the original monitoring point has increased after the excavation, and the closer the point is to the center of the right tunnel, the larger the value is. The maximum settlement value in the simulation was 22.8 mm.

**Figure 13:**
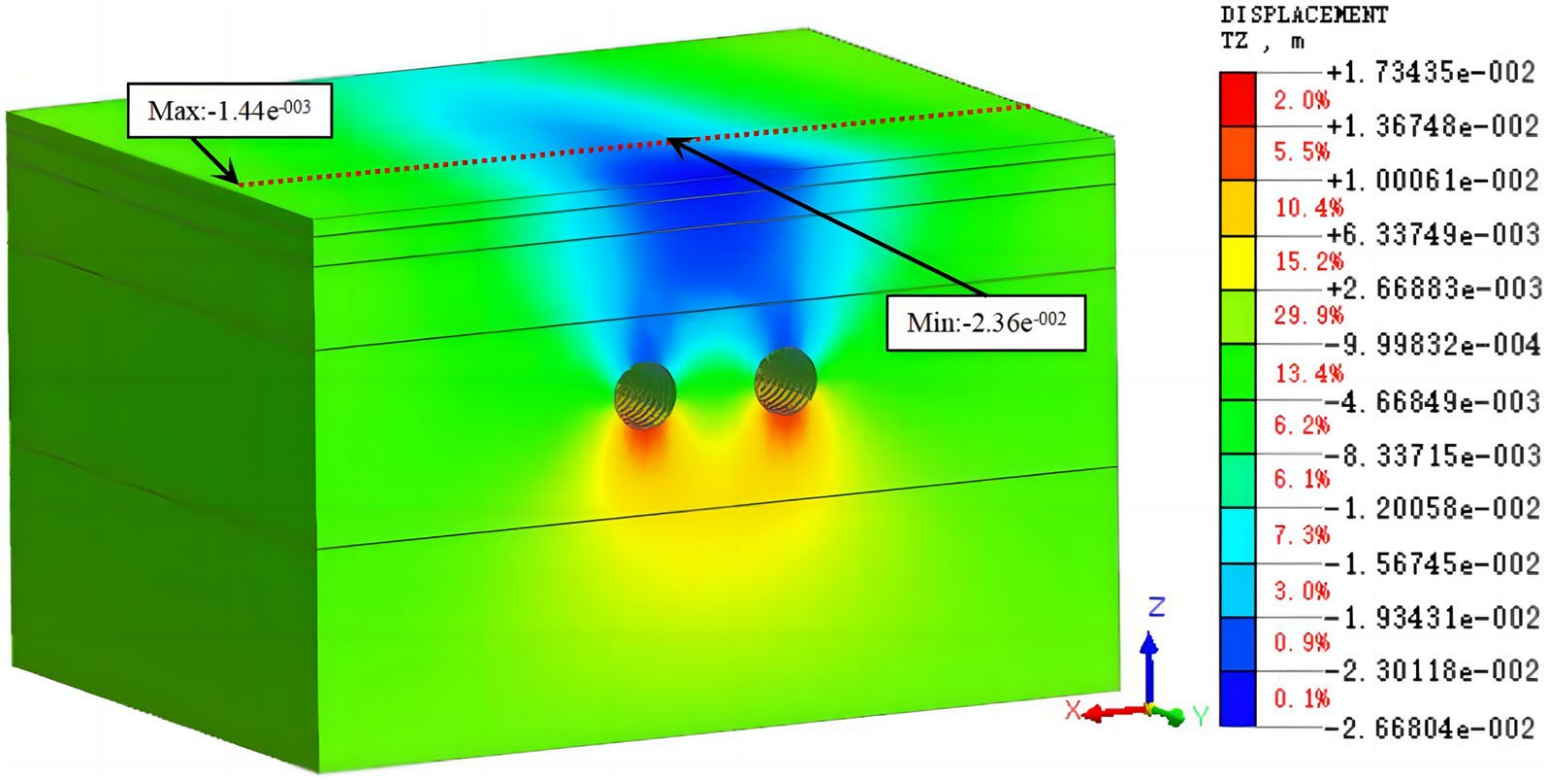
The cloud diagram of the vertical displacement for working condition 2 (double-line excavation).

Working condition 2: In numerical simulations, the simultaneous construction of both tunnels is the simplest and fastest strategy; thus, this modeling method is widely used. However, it does not consider the influence of the construction process on the surface settlement. This method is not used in the actual project but is used in this study to compare it with the other working conditions. The cloud diagram of the vertical displacement of the tunnel excavation is shown in Fig. 13.

**Figure 14:**
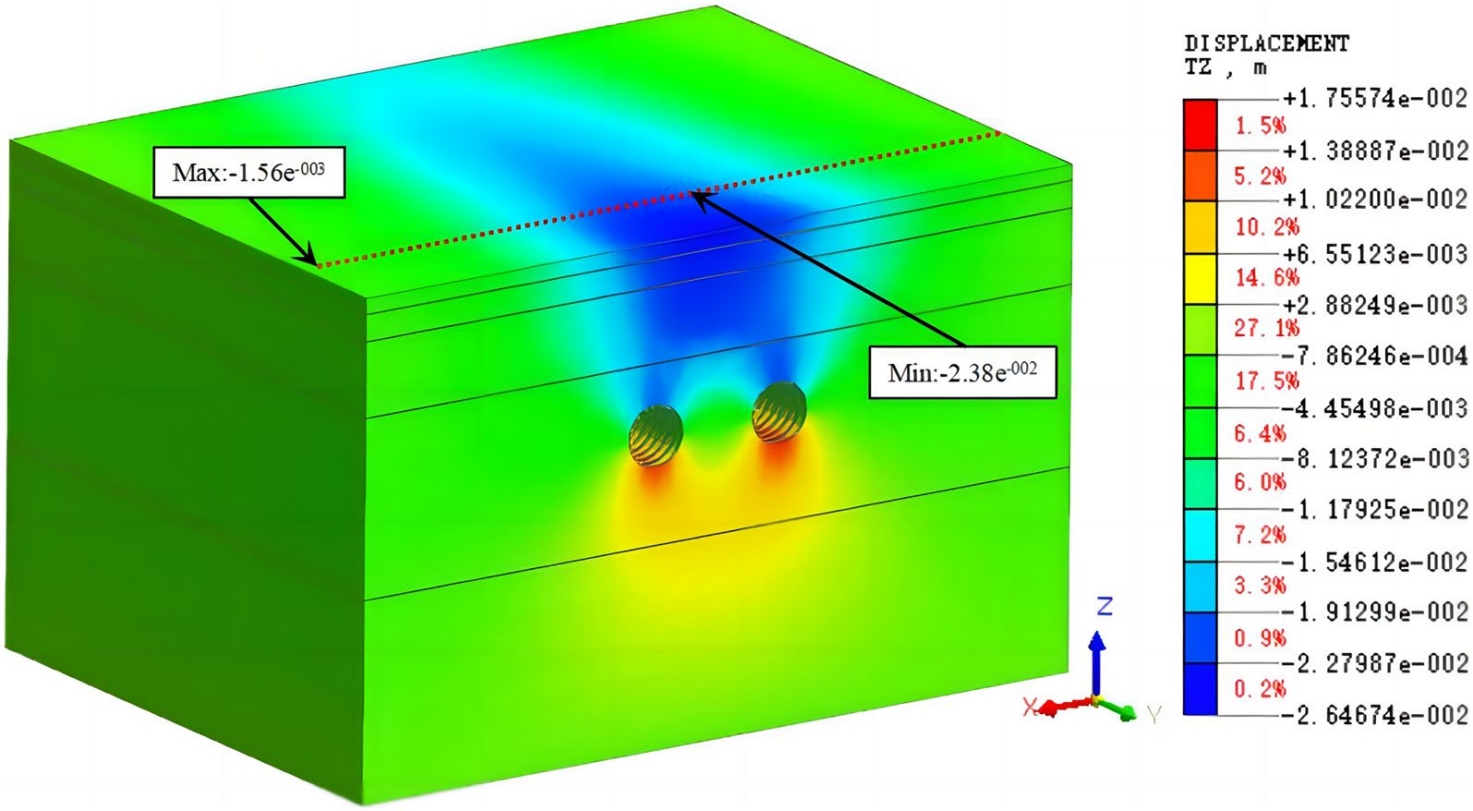
The cloud diagram of the vertical displacement for working condition 3 (asynchronous excavation).

Working condition 3: This working condition simulated the asynchronous excavation of the left and right lines. The spacing of the asynchronous excavation of the left and right lines was set to 20 rings initially. The right line was excavated after the left line had been excavated for 24 m (4 times the diameter of the hole). In actual engineering projects, section spacing during asynchronous excavation of the left and right lines is not fixed. This model simulated the asynchronous excavation of the double-line tunnel after the completion of the 100th ring of the left line. The cloud diagram of the vertical displacement is shown in Fig. 14.

**Figure 15:**
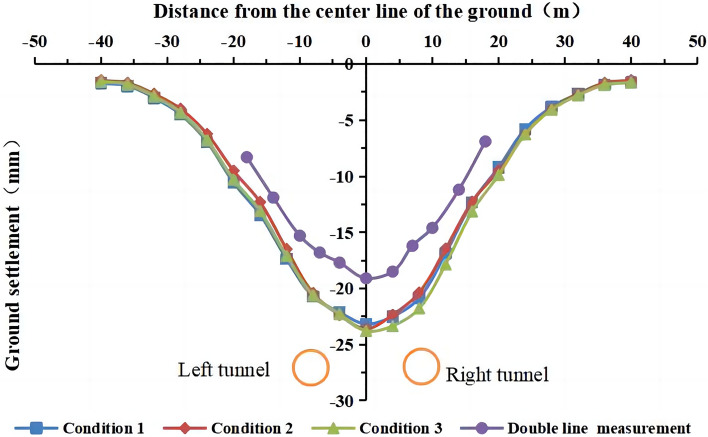
Comparison of the simulated and measured surface settlement for the three working conditions.

Figure 15 shows the simulated and measured surface settlement after the completion of the double-line excavation for the three working conditions at y = 50 m.

Figures 13, 14, 15 show that the simulated values of the surface settlement are consistent with the measured values for the three working conditions. The maximum surface settlement values obtained from the simulation are −23.2 mm, −23.6 mm, and −23.8 mm for the three working conditions, respectively. The values are similar for the three conditions. However, the measured settlement is 19.1 mm smaller than the simulated settlement. The measured and simulated surface settlement curves exhibit the same trend and have a V shape.

The models of working conditions 1 and 3 simulated the excavation of the tunnel in different stages of the project. The model of working condition 1 can also be used to simulate the excavation and construction of the other side after the first tunnel has been completed. During the construction of a double-line tunnel, it is necessary to consider construction risks and costs related to uncontrollable factors, such as increased ground disturbance caused by the simultaneous construction of the left and right lines, the mechanical stability of the shield machine, and the difference in the technical expertise of the construction team. Although the simultaneous excavation of the two tunnels in the same direction (working condition 2) is not used in actual engineering, the modeling mode can be used to study the influence of the stratum parameters and other factors on the surface settlement. Working condition 2 is easier to implement than working condition 1 and working condition 3. Condition 2 is widely used in numerical simulations that do not consider asynchronous excavation and construction. The simulation of working condition 1 is the most consistent with the shield excavation 120 m before the tunnel in this engineering example. Moreover, the comparative analysis shows that the construction sequence in the numerical simulation has no significant influence on the surface settlement. In working condition 1, we simulated the single-line excavation first, followed by the double-line excavation to compare the results. Therefore, we use the model of working condition 1 in the subsequent analysis. The curve of the simulated settlement is smoother than that of the measured settlement. The analysis shows that the numerical simulation result is the ideal value under the assumed conditions. However, the factors affecting the surface settlement are complex in the real project. Mudstone is characterized by uneven weathering, and there are differences in the mechanical parameters of the rock and soil. The different layers are assumed to be continuous and have a uniform medium. The adjustment of the dynamic parameters during construction and the operation of heavy vehicles affect the monitoring results of surface subsidence.

## Sensitivity analysis of surface subsidence parameters

### Sensitivity of formation parameters

In this case study, shield construction is conducted in a strongly weathered mudstone layer. Weathered mudstone softens rapidly when it contacts water and easily disintegrates when it loses water. This stratum type typically exhibits uneven weathering. Differences in the physical and mechanical properties affect the surface settlement during shield construction. We analyzed the effects of the deformation modulus, cohesion, and internal friction angle of the strongly weathered mudstone layer on the surface settlement in working condition 1.

#### The influence of the deformation modulus on surface settlement

**Figure 16:**
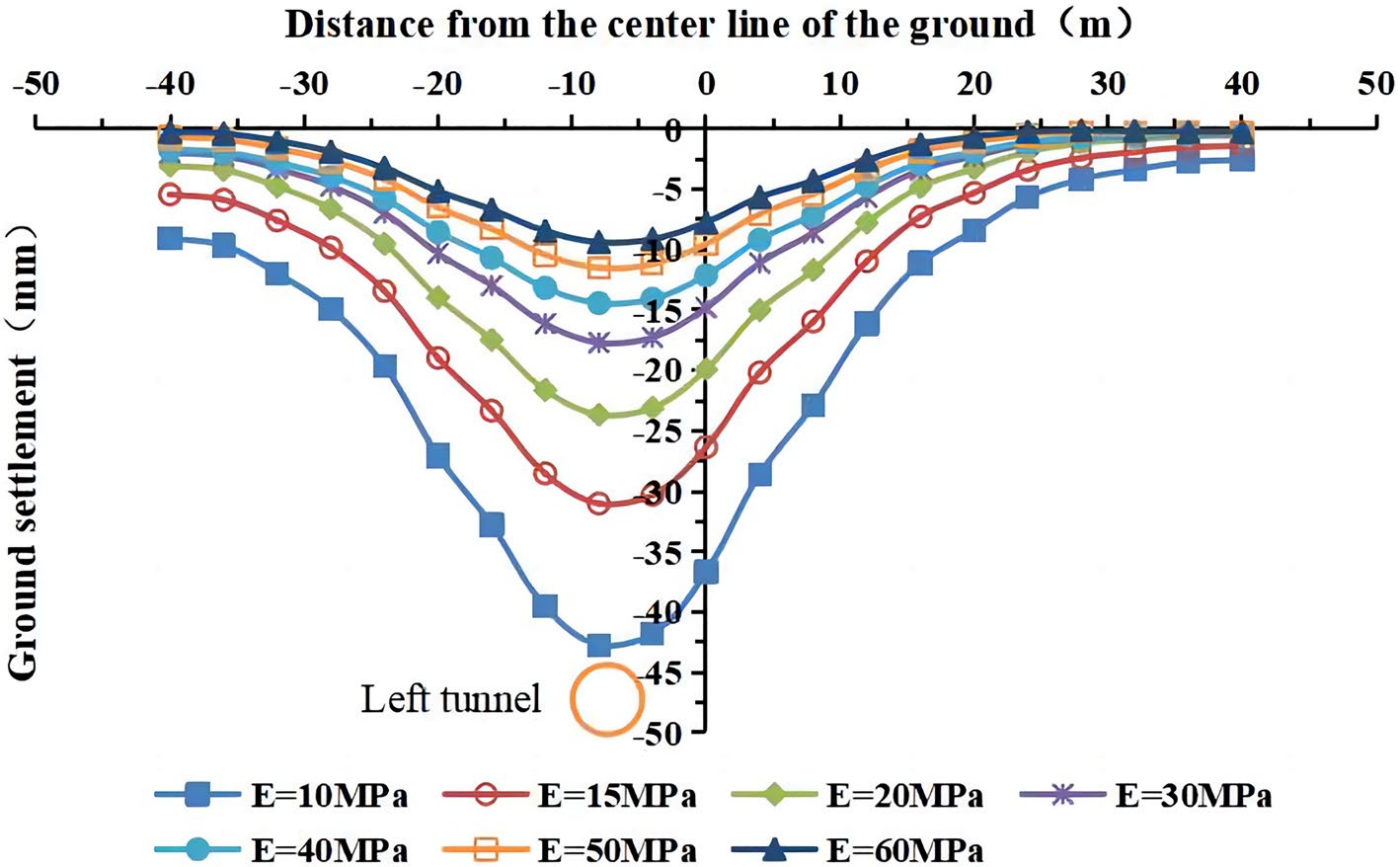
Surface settlement during single-line excavation for different deformation modulus values.

**Figure 17:**
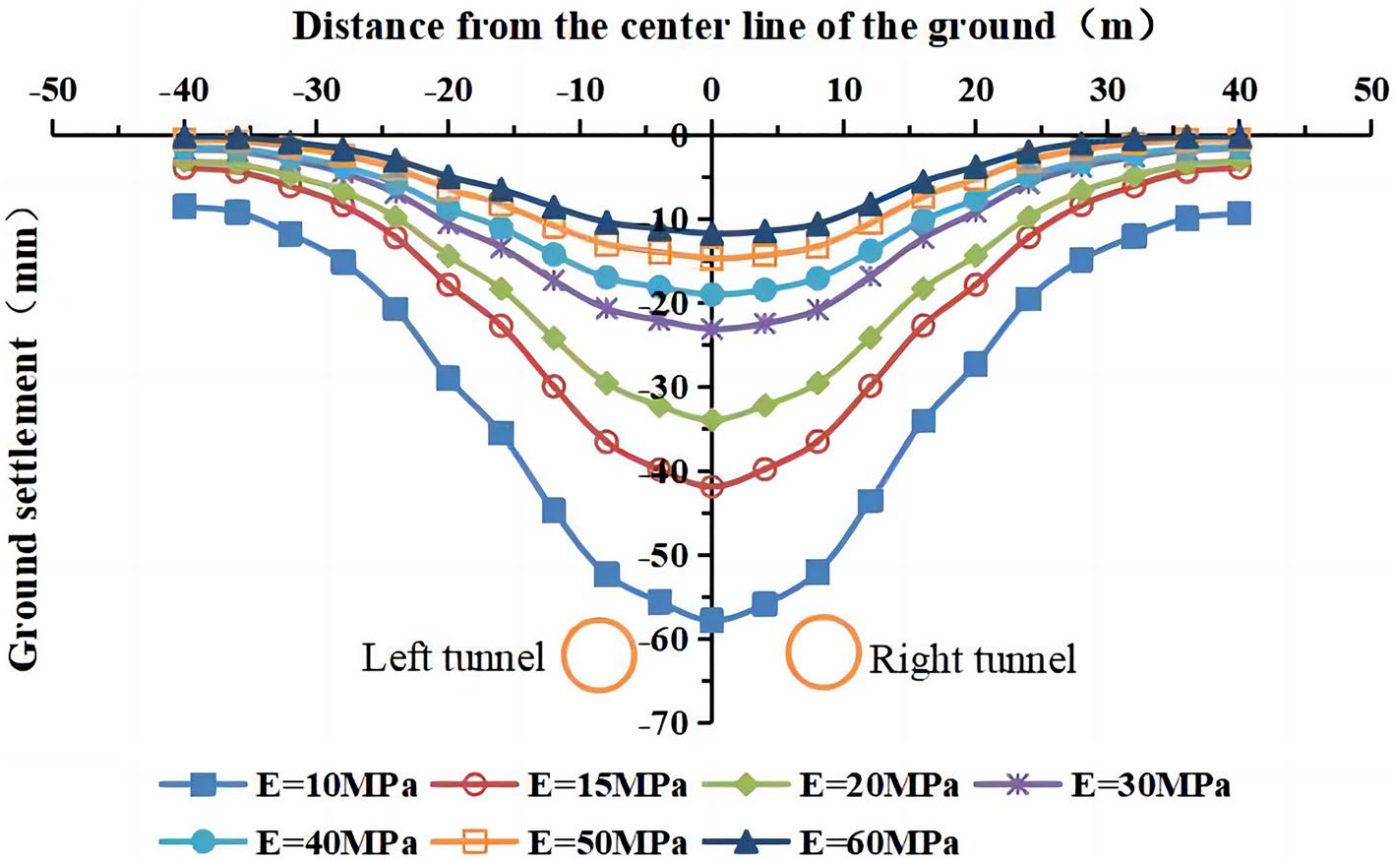
Surface settlement during double-line excavation for different deformation modulus values.

**Figure 18:**
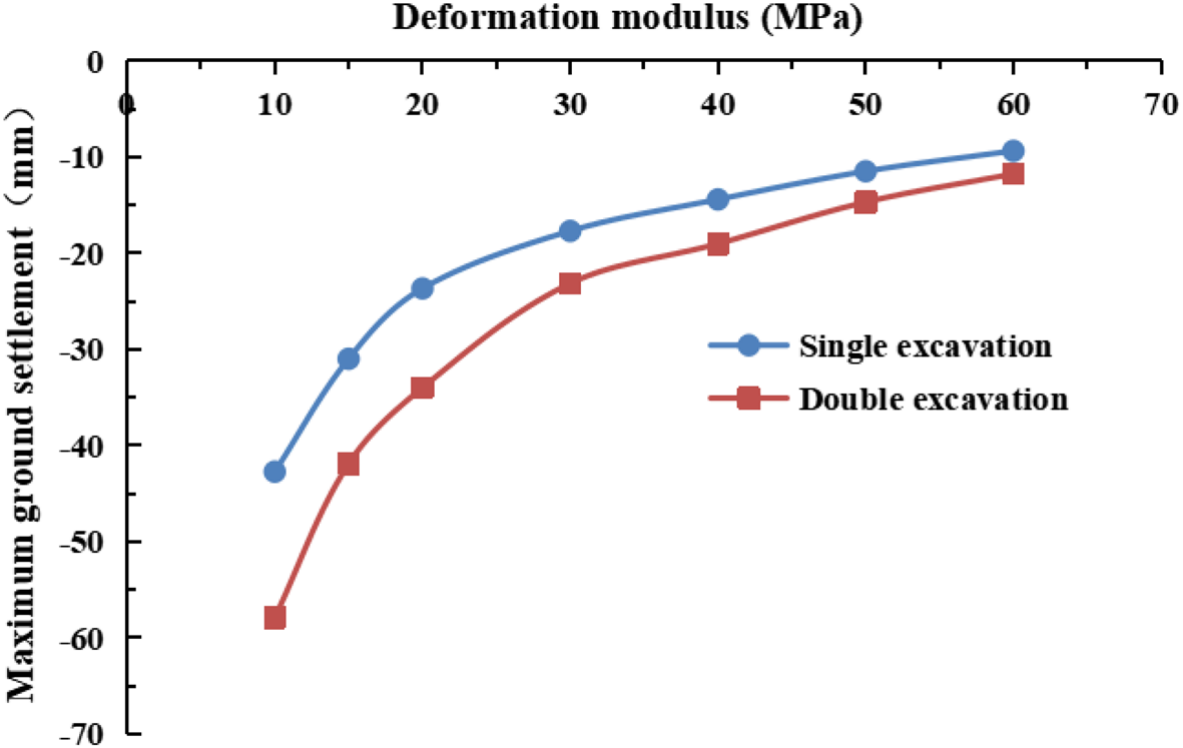
The maximum surface settlement during single-line and double-line excavation for different deformation modulus values.

The values of the deformation modulus of the strongly weathered mudstone layer were E = 10 MPa, 15 MPa, 20 MPa, 30 MPa (actual value), 40 MPa, 50 MPa, and 60 MPa in the numerical simulations. The lateral surface settlement values in the y = 50 m section for different values of the deformation modulus are shown in Figs. 16 and 17. The maximum surface settlement during single-line and double-line excavation is shown in Fig. 18.

As the deformation modulus of the strongly weathered mudstone layer increases, the surface settlement decreases during single-line and double-line excavation. A significant decrease in the surface settlement is observed as the deformation modulus increases from 10 to 30 MPa. For example, the maximum surface settlement during double-line excavation decreases from −57.9 to −23.2 mm, a decrease of 34.7 mm. As the deformation modulus increases, its influence on the surface settlement weakens. As the deformation modulus increases from 30 to 60 MPa, the maximum surface settlement during double-track excavation decreases from −23.2 to −11.8 mm, a decrease of 11.4 mm. These results indicate the significant influence of the deformation modulus on surface subsidence. Therefore, the uneven weathering of the mudstone layers in the formation should be considered.

#### The effect of cohesion on surface settlement

**Figure 19:**
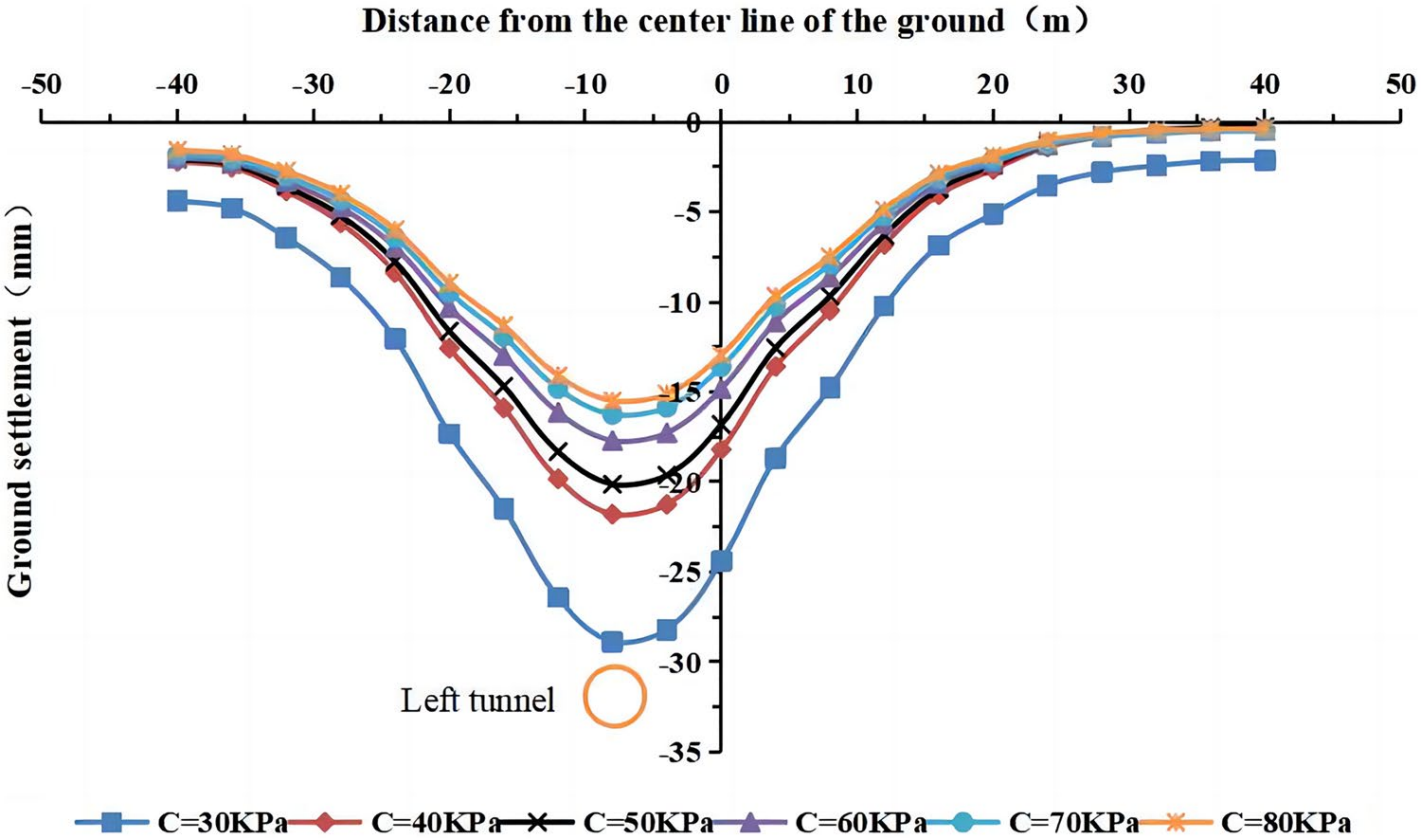
Surface settlement during single-line excavation for different cohesion values.

**Figure 20:**
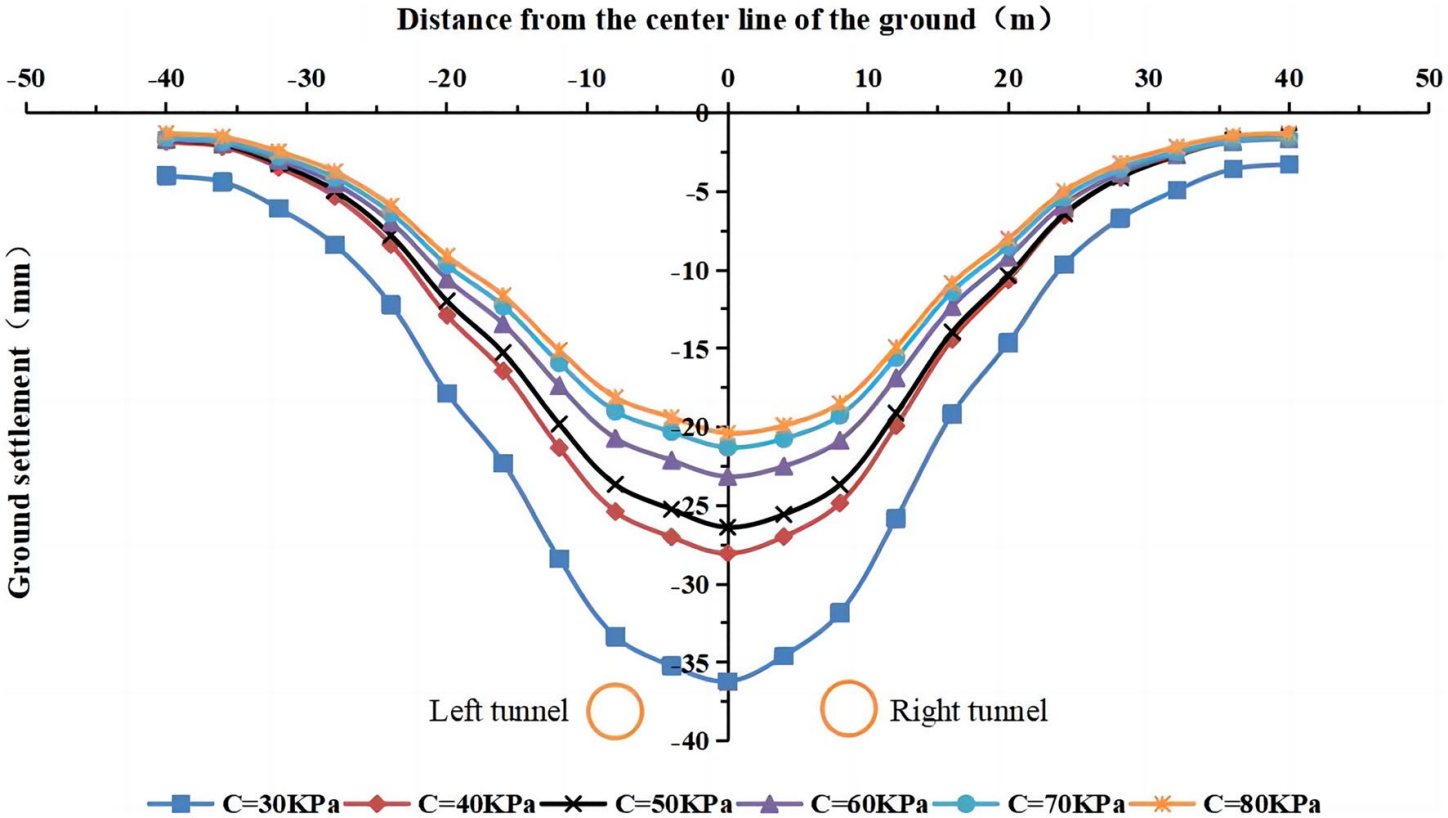
Surface settlement during double-line excavation for different cohesion values.

**Figure 21:**
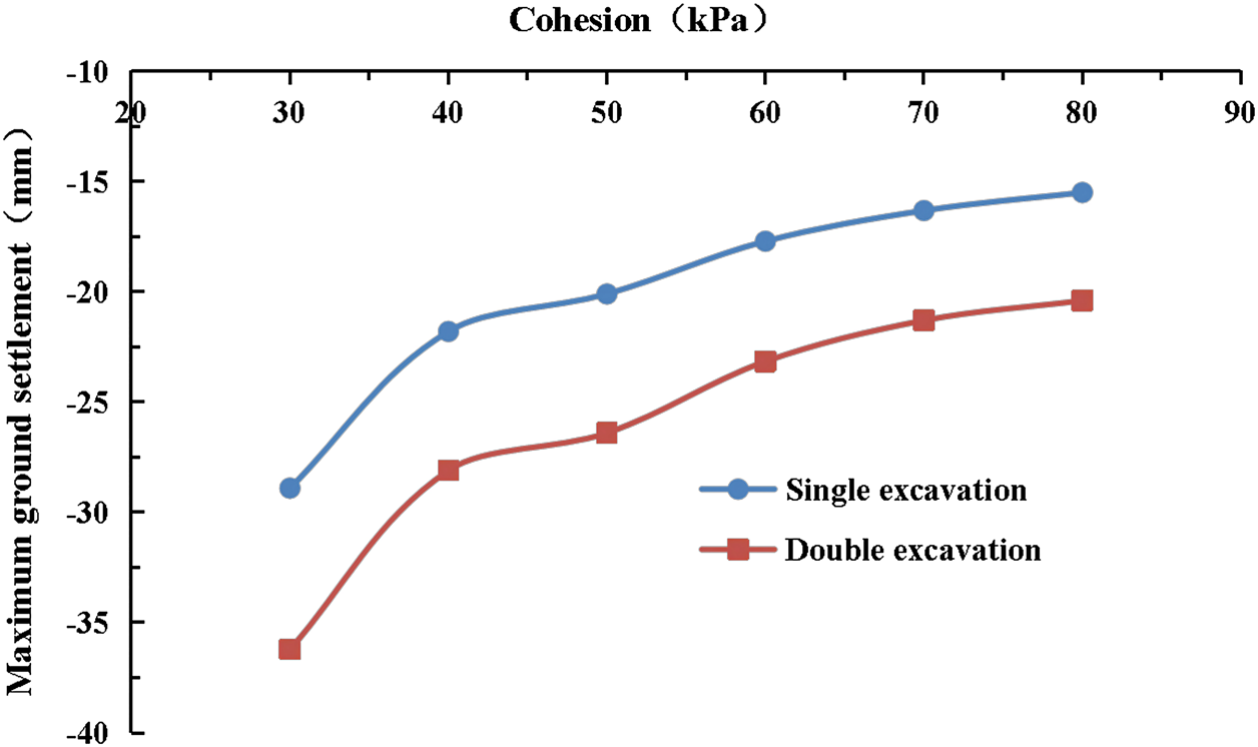
The maximum surface settlement during single-line and double-line excavation for different cohesion values.

The values of the cohesive force of the strongly weathered mudstone layer were C = 30 kPa, 40 kPa, 50 kPa, 60 kPa (actual value), 70 kPa, and 80 kPa in the numerical simulation. The lateral surface settlement values for different cohesive forces are shown in Figs. 19 and 20. The maximum surface settlement during single-line and double-line excavation is shown in Fig. 21.

The largest surface settlement value during single-line excavation (28.9 mm) and double-line excavation (36.2 mm) is observed at a cohesion value of C = 30 kPa. The smallest values occur at 80 kPa, i.e., 15.5 mm for single-line excavation and 20.4 mm for double-line excavation. The maximum value of the surface settlement decreases with an increase in cohesion, and an inflection point occurs at C = 40 kPa. The cohesion has a more pronounced influence on the surface settlement at values of less than 40 kPa. As shown in Fig. 21, the maximum surface settlement decreases with an increase in cohesion. However, the rate of change is high as C increases from 30 to 40 kPa and low when the cohesion exceeds 40 kPa.

#### The influence of the internal friction angle on surface settlement

**Figure 22:**
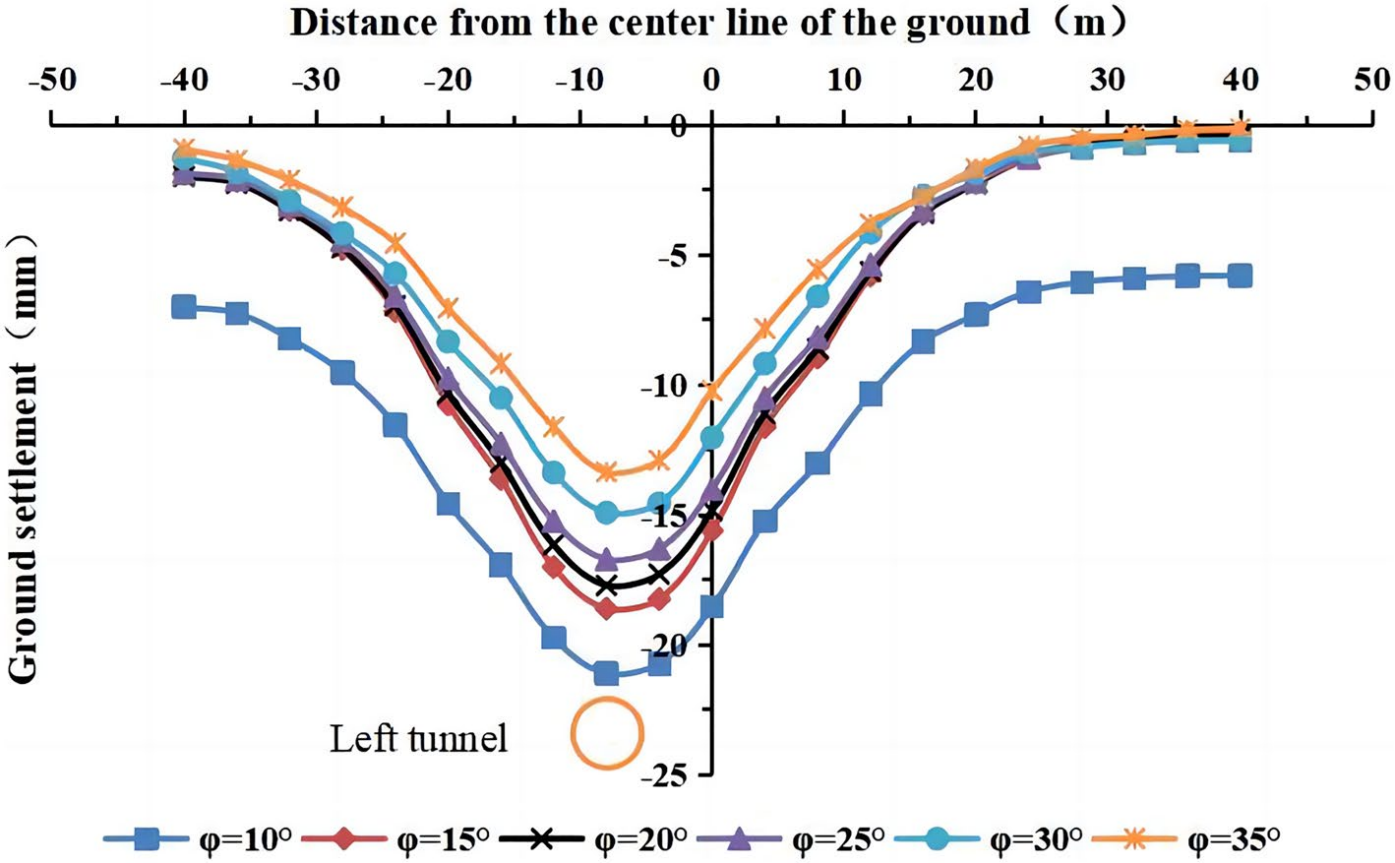
Surface settlement during single-line excavation for different internal friction angles.

**Figure 23:**
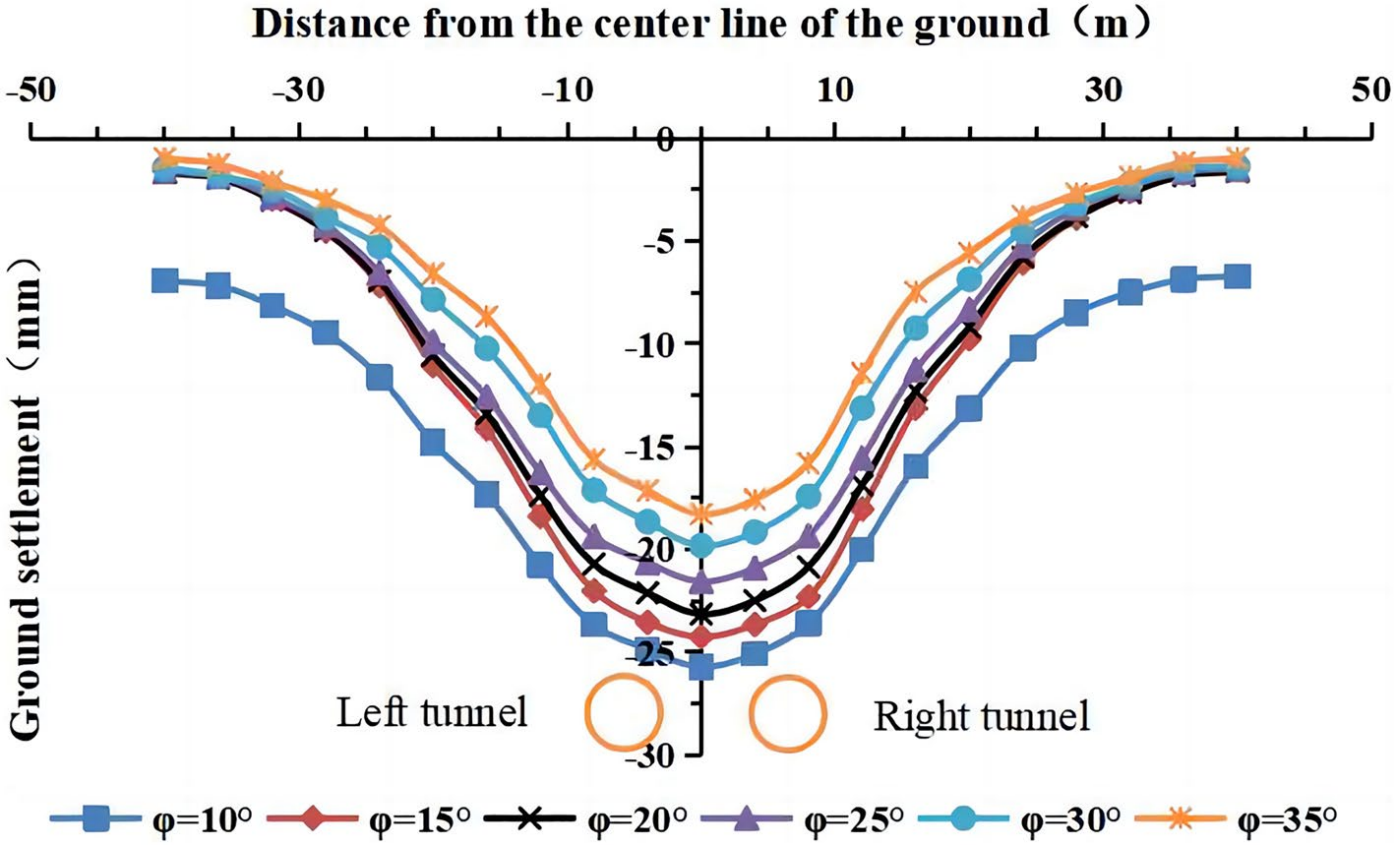
Surface settlement during double-line excavation for different internal friction angles.

**Figure 24:**
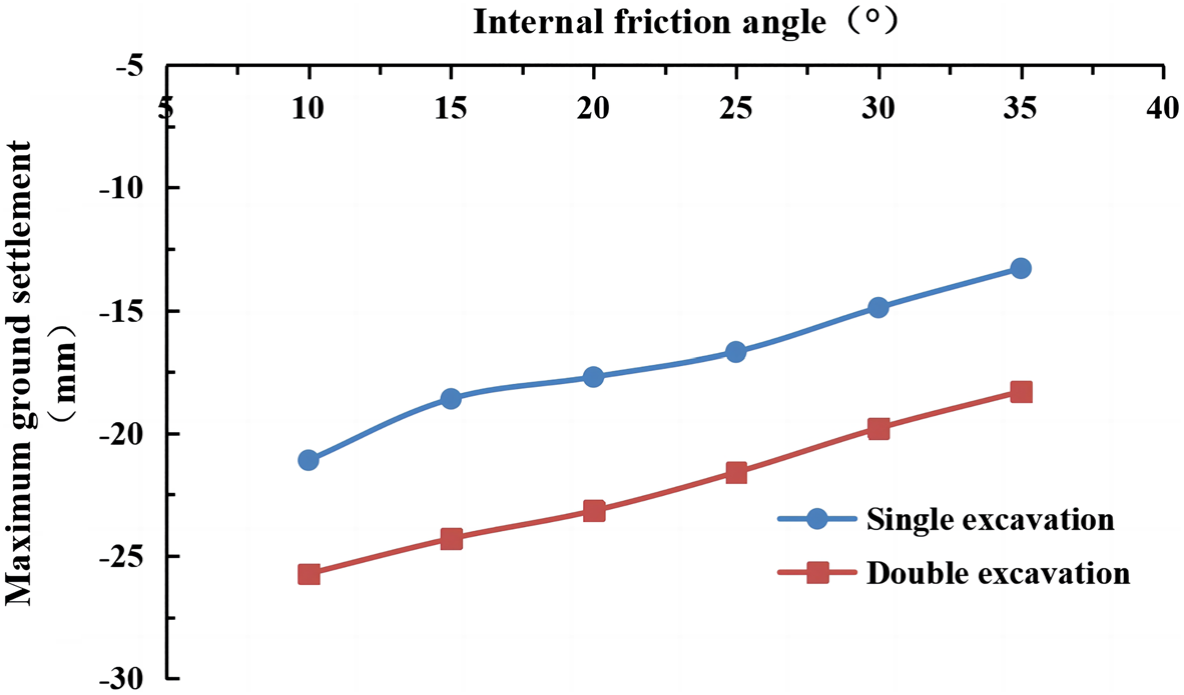
The maximum surface settlement during single-line and double-line excavation for different internal friction angles.

The internal friction angles of the strongly weathered mudstone layer were φ = 10°, 15°, 20°, 25° (actual value), 30°, and 35° in the numerical simulation. The lateral surface settlement values for different internal friction angles are shown in Figs. 22 and 23. The maximum surface settlement during single-line and double-line excavation is shown in Fig. 24.

The maximum surface settlement occurs at an internal friction angle of 10° during single-line excavation (21.1 mm) and double-line excavation (25.7 mm). The smallest values occur at 35°, i.e., 13.3 mm for single-line excavation and 18.3 mm for double-line excavation. The settlement trough is the narrowest at an internal friction angle of φ = 35°. The maximum surface settlement decreases, and the width of the settlement trough increases with an increase in the internal friction angle.

### Sensitivity of tunnel design parameters

#### The influence of the buried depth of the tunnel axis on the surface settlement

**Figure 25:**
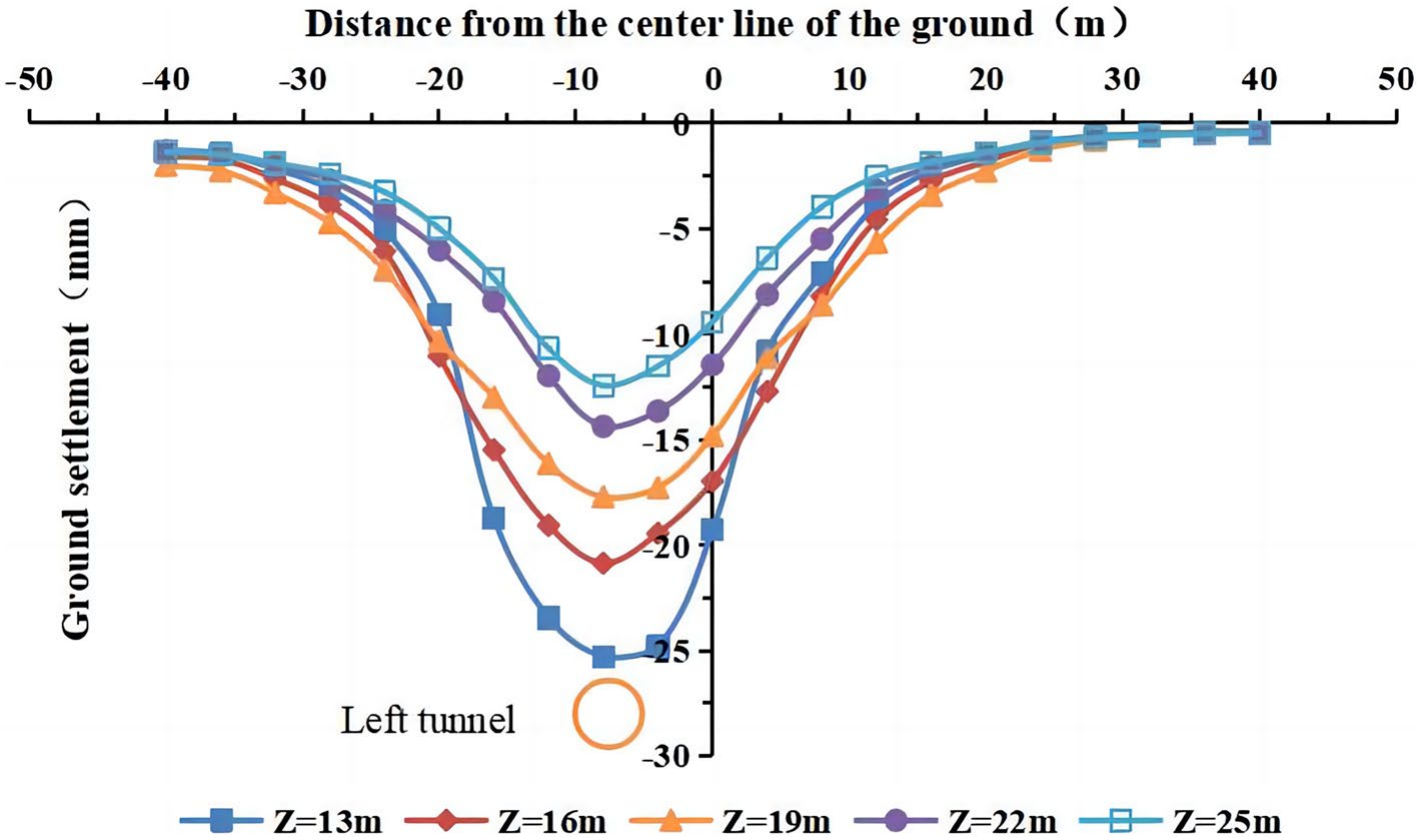
Surface settlement during single-line excavation for different buried depths.

**Figure 26:**
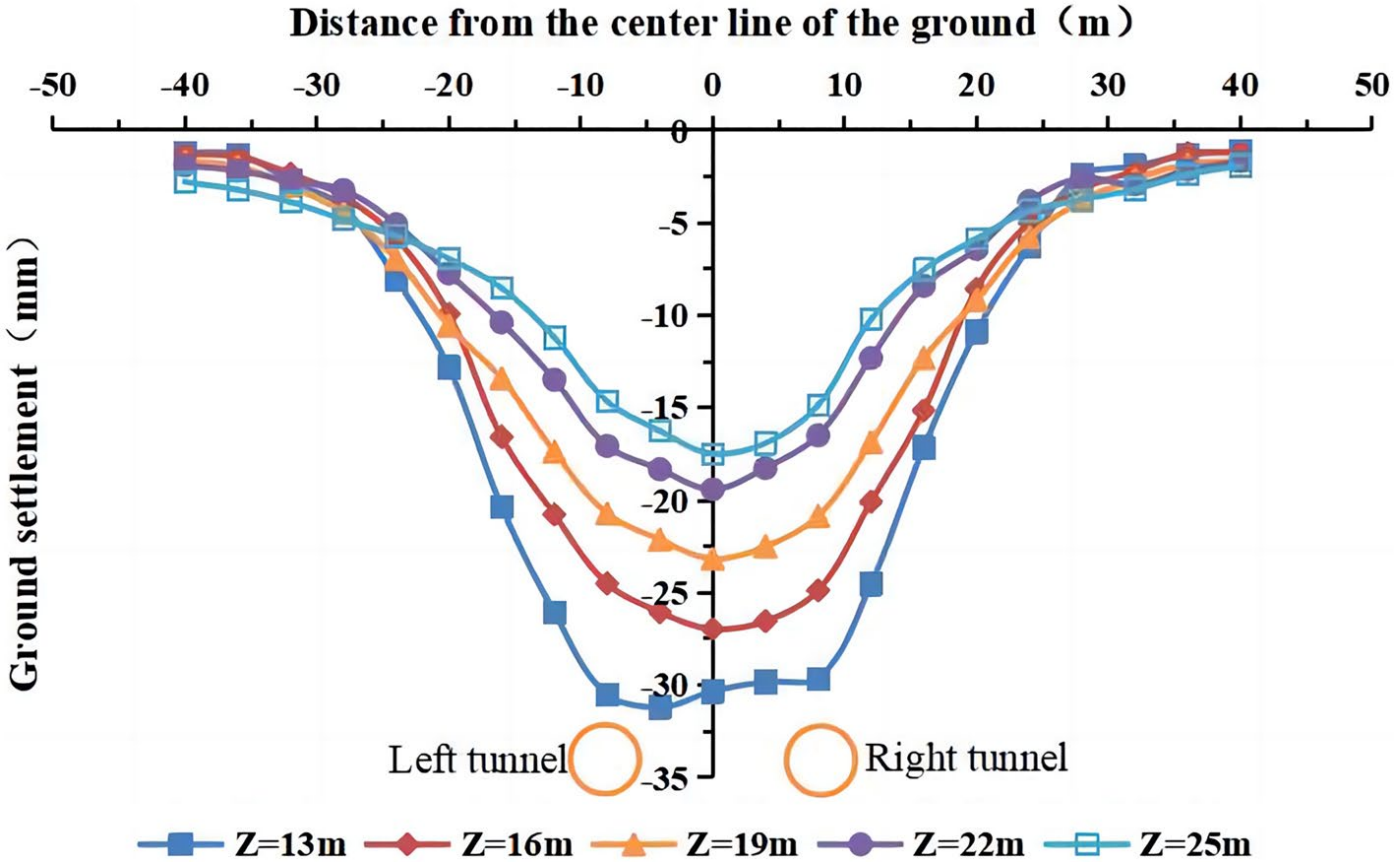
Surface settlement during double-line excavation for different buried depths.

The buried depth of the tunnel axis was 13 m, 16 m, 19 m, 22 m, and 25 m, and the other parameters remained unchanged. The lateral settlement curves of the ground surface after the completion of the single and double-line excavation at different buried depths are shown in Figs. 25 and 26.

The maximum surface settlement decreases, and the width of the settlement trough increases with an increase in the buried depth of the tunnel. The maximum surface settlement during single-line excavation (−25.3 mm) and double-line excavation (−31.2 mm) occur at a buried depth of 13 m. The surface settlement curve of the single-line excavation shows an approximately normal distribution as the buried depth of the tunnel changes, and the maximum surface settlement occurs at the center of the tunnel. The surface settlement curve of the double-line excavation shows an approximately normal distribution at a buried depth of 16–25 m. At a buried depth of 13 m, the surface settlement curve has two peaks at the centers of the left and right tunnels. Thus, the buried depth of the tunnel affects the surface settlement and the width of the settlement trough, and two peaks occur in the curve at the lowest buried depth in this simulation of the double-line excavation. As shown in Figs. 25 and 26, the maximum value of the surface settlement decreases with an increase in the tunnel’s burial depth, and the surface settlement on both sides of the tunnel shows an increasing trend, indicating that the influence range of the surface settlement increases, i.e., the width of the surface settlement trough increases.

#### The influence of the distance between the left and right centerlines of the tunnel on the surface settlement

**Figure 27:**
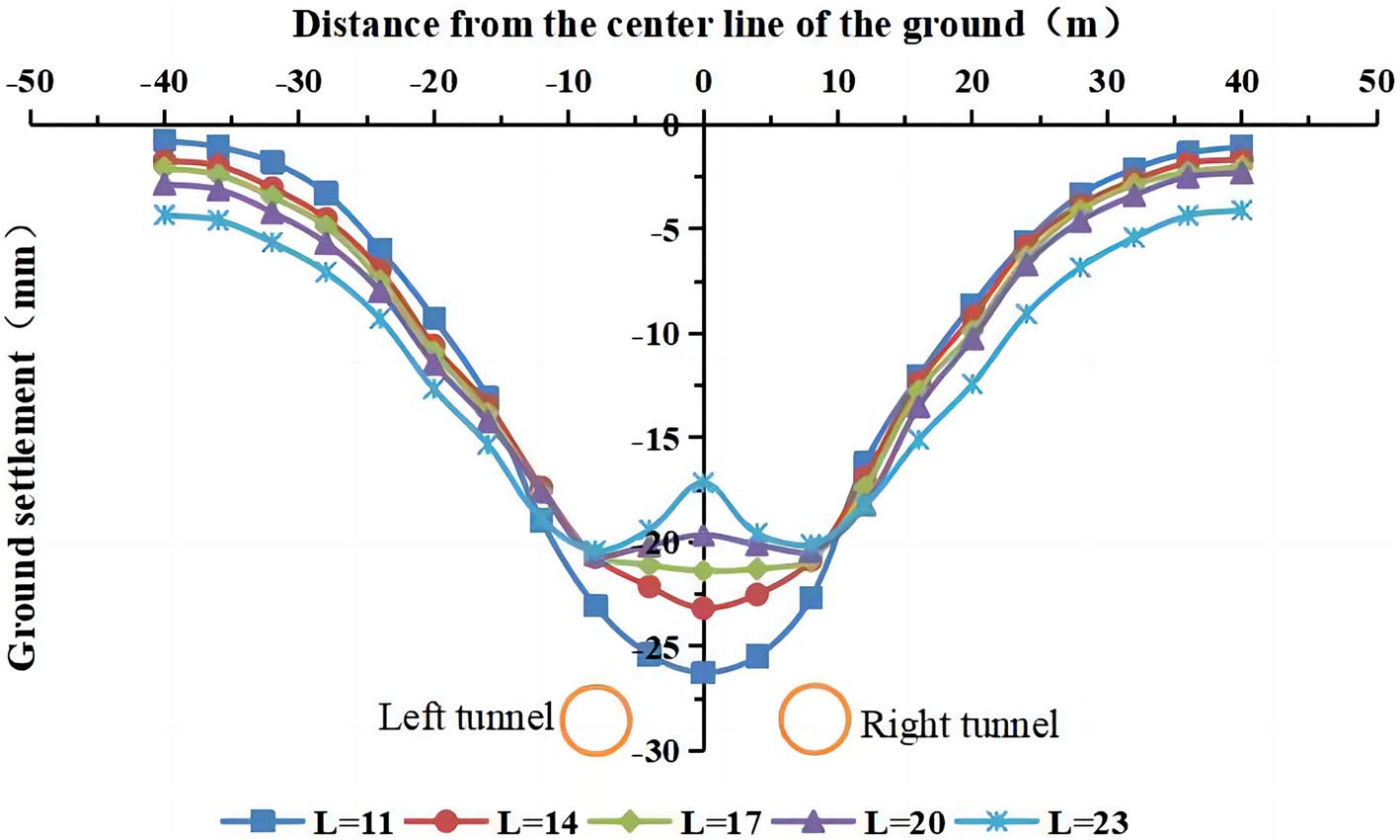
The surface settlement for different distances between the two tunnels.

The distances between the left and right center lines of the tunnel were 11 m, 14 m (actual value), 17 m, 20 m, and 23 m, and the other parameters remained unchanged. The surface settlement for different distances between the two tunnels is shown in Fig. 27.

The maximum surface settlement decreases with an increase in the distance between the two tunnels. At a distance of 11 m, the maximum surface settlement is −26.3 mm at the center between the two tunnels. As the distance increases to 23 m, the maximum surface settlement decreases to −20.4 mm. As the distance between the two tunnels increases, the width of the settlement trough increases, and the settlement curve changes from a V shape with an approximately normal distribution to a W shape with two peaks. The change in the shape of the surface settlement curve indicates that the mutual influence between the two tunnels decreases as the distance between the tunnels increases. In this working condition, the distance exceeds 20 m (> 3 D, where D is the diameter of the tunnel). The larger the spacing, the more pronounced the bimodal shape of the curve is.

### Tunneling parameters of shield construction

#### The influence of the shield machine’s thrust on surface settlement

**Figure 28:**
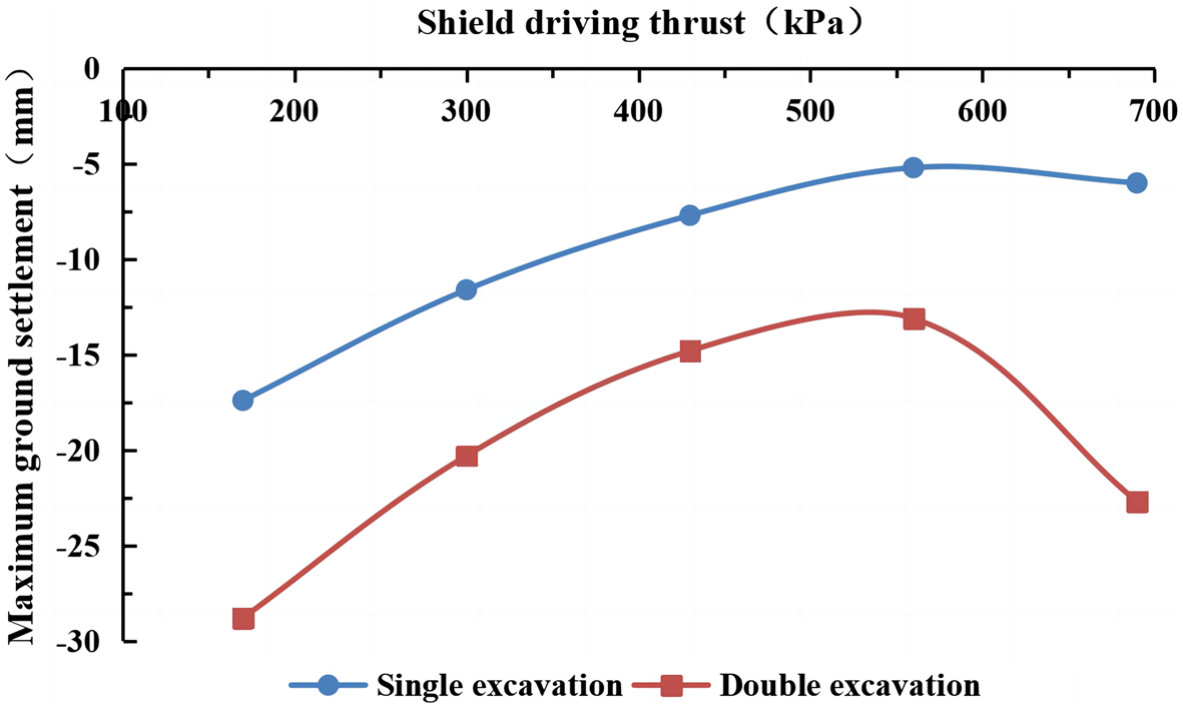
The maximum surface subsidence for different thrust forces of the shield machine.

The actual thrust of the shield machine is 9000–13000 kN. The thrust was simplified in the numerical simulation as a uniformly distributed load on the excavation surface of 300–430 kPa. Thrust values of 170 kPa, 300 kPa, 430 kPa, 560 kPa, and 690 kPa were used in the simulation, and the other parameters remained unchanged. The surface settlement for different thrust forces of the shield machine is shown in Fig. 28. The maximum surface subsidence occurred at monitoring point A (40, 30, 0).

**Figure 29:**
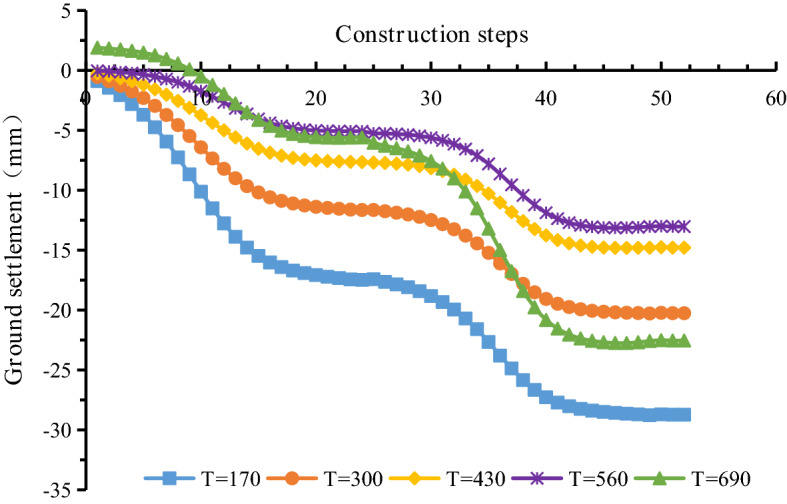
The surface settlement for different thrusts of the shield machine.

The surface settlement values at monitoring point A (40, 30, 0) for different thrusts are shown in Fig. 29.

Figure 28 indicates that the surface settlement decreases as the thrust of the shield machine increases from 170 to 560 kPa. The maximum surface settlement decreases from 17.4 to 5.2 mm during single-line excavation and from 28.4 mm to 13.1 during double-line excavation. When the shield tunneling pressure is 690 kPa, there is no need to maintain the balance between the earth pressure of the shield machine and the earth pressure of the excavation face. The soil exhibits large deformation. The ground surface in front of the shield machine rises locally. After the shield machine passes, the ground surface begins to settle and stabilizes. The double-line tunnel shield machine results in a significant ground disturbance. Therefore, a significant change in the surface settlement is observed at a thrust of 690 kPa, and the effect is more pronounced for the double-line excavation. Figure 29 shows that a high thrust causes surface uplift in the initial stage of the left-line excavation, which is consistent with the actual results. After the excavation of the right line has started, a high thrust accelerates the increase in surface settlement. Thus, a suitable thrust should be chosen to reduce surface settlement. During the construction of the double-line tunnel, the shield machine significantly disturbs the stratum, especially in the middle of the left and right tunnels. Therefore, excessive thrust results in surface uplift and may cause greater surface settlement during double-line construction.

#### The influence of the shield grouting quality on surface settlement

**Figure 30:**
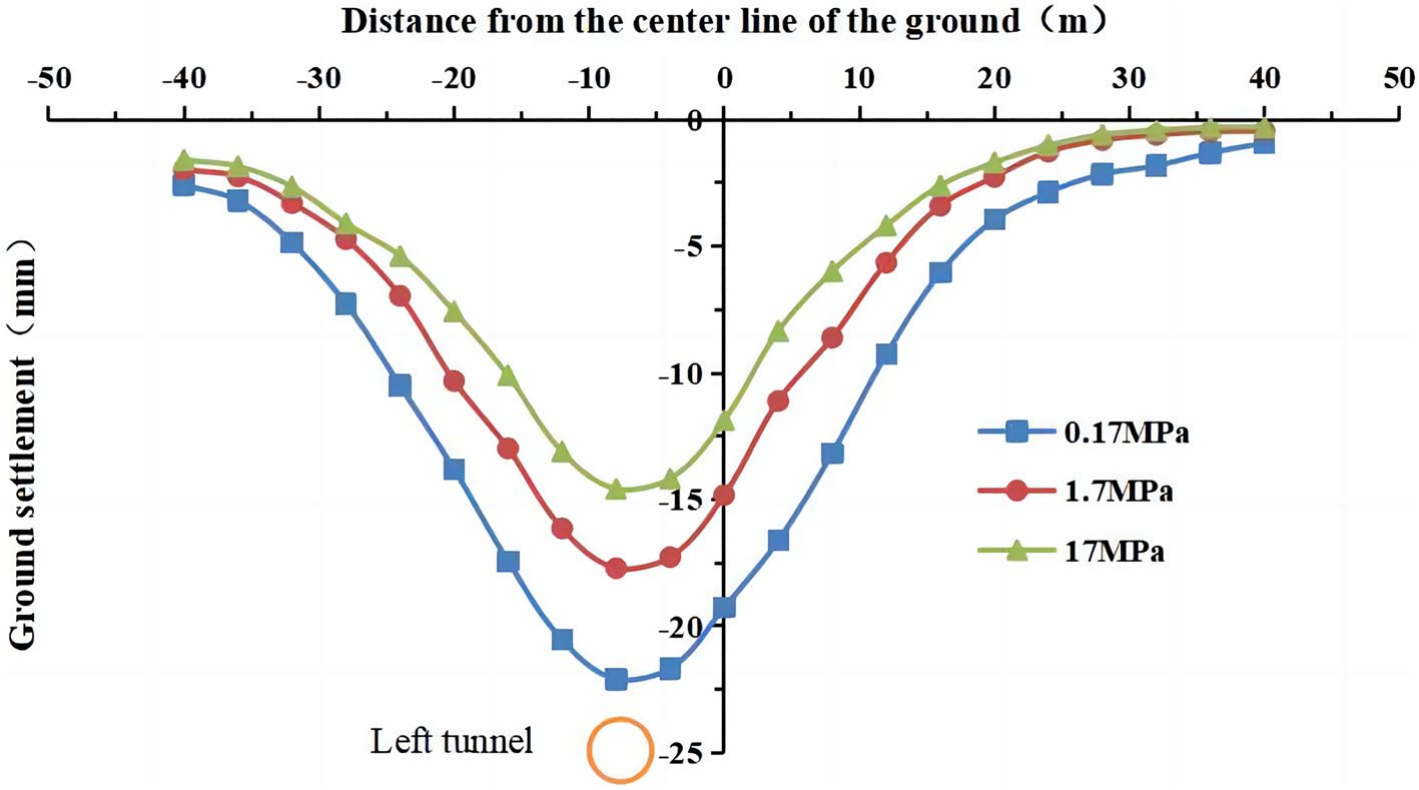
Surface settlement during single-line excavation for different deformation modulus values of the grouting layer.

**Figure 31:**
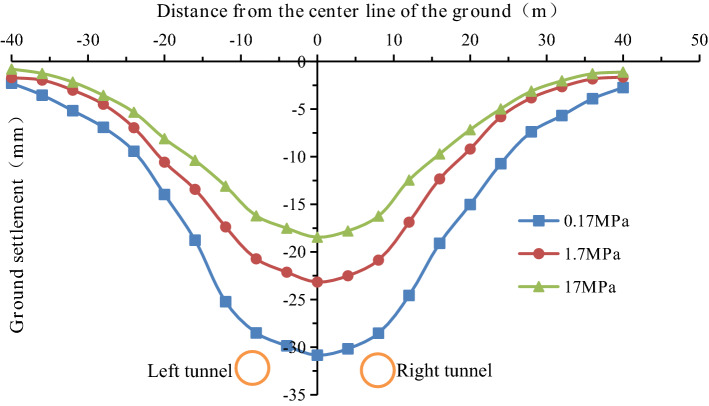
Surface settlement during double-line excavation for different deformation modulus values of the grouting layer.

Many factors affect the grouting quality, such as the ratio of the grouting liquid, the grouting pressure, and the grouting duration time. In this numerical simulation, the deformation modulus of the grouting layer is used as a proxy of the grouting quality. The deformation modulus of the grouting layer was E = 0.17 MPa, 1.7 MPa, and 17 MPa in the numerical simulation. The settlement values at the monitoring point of the profile at y = 50 m are shown in Fig. 30 and Fig. 31.

The maximum surface settlement values during single-track excavation (22.1 mm) and double-track tunnel excavation (30.8 mm) occur at a deformation modulus of 0.17 MPa. The minimum surface settlement values during single-track excavation (14.6 mm) and double-track tunnel excavation (18.5 mm) occur at a deformation modulus of 17 MPa. The surface settlement decreases with an increase in the deformation modulus of the grouting layer, i.e., the higher the quality of the grouting layer, the smaller the surface settlement is.

### The influence of the section spacing between the left and right lines during asynchronous excavation on surface settlement

**Figure 32:**
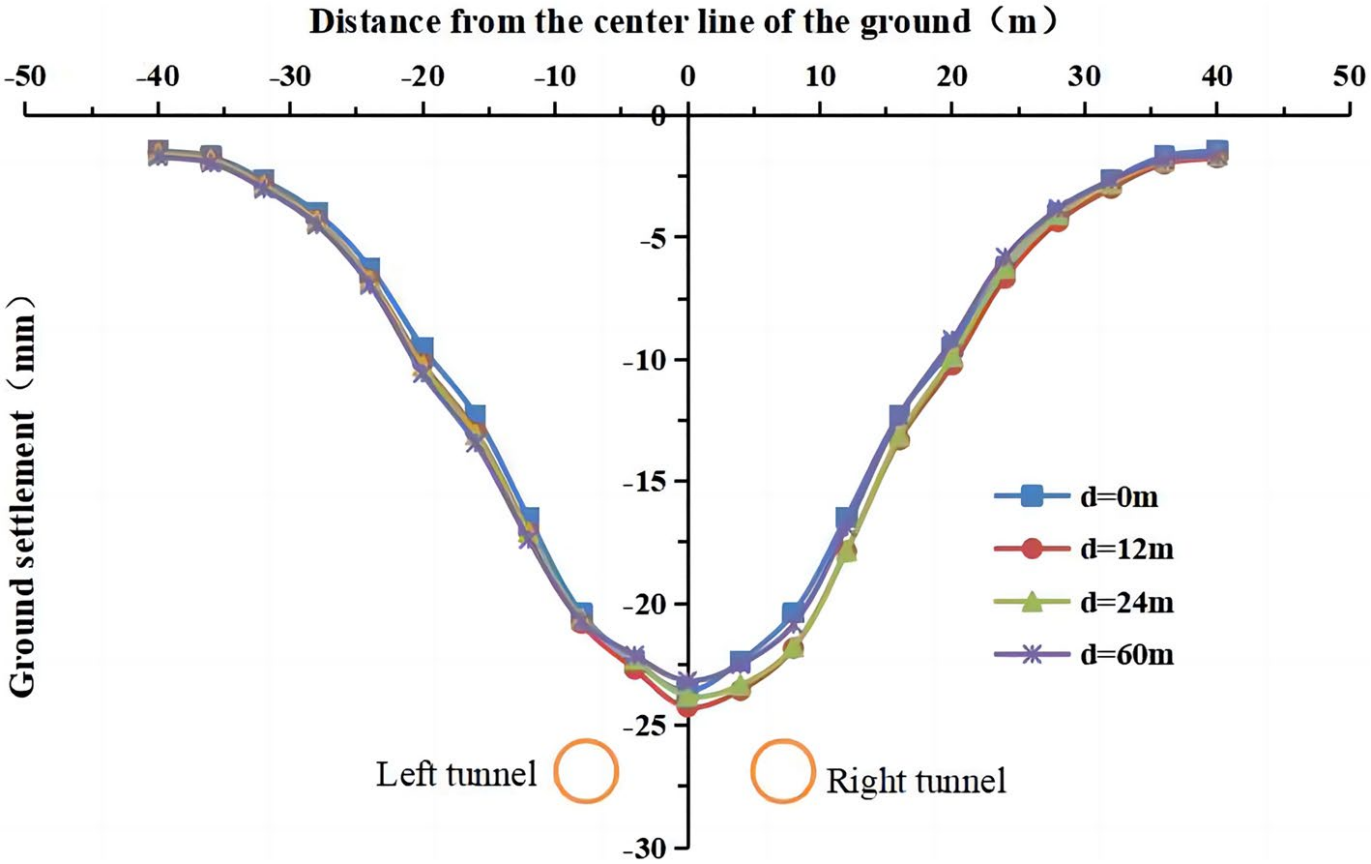
Surface settlement for different section spacings between the left and right lines.

The model of working condition 3 was used to simulate the influence of the section spacing between the left and right lines (12 m (2D) and 24 m (4D), where D is the diameter of the tunnel) during asynchronous excavation on surface settlement. In working condition 1, the left line excavation was completed before the right line excavation was started, which was equivalent to a section spacing of d = 60 m. The distance was d = 0 m in the simultaneous excavation of the left and right lines (working condition 2). The surface settlement for different distances section spacings between the left and right lines is shown in Fig. 32.

The trends of the lateral surface settlement are similar for different section spacings during asynchronous excavation, and the curves show an approximately normal distribution. The maximum surface settlement is the largest (24.2 mm) at a section spacing of d = 12 m and the smallest (23.2 mm) at d = 60 m. A section distance of d = 0 m has the largest impact on the surface settlement (23.6 mm). However, the differences between the maximum surface settlement values are very small for different section spacings, indicating that the section spacing is not a highly influential factor on the surface settlement during asynchronous excavation of double-line shield construction.

## Conclusion

We conducted finite element numerical simulations of a double-line tunnel construction project in the soft mudstone area of Changchun City and obtained field monitoring data to investigate the influence of various factors on the surface settlement. The following conclusions were drawn:

1. The numerical simulation results of the surface settlement during shield construction were consistent with the monitoring results, indicating the reliability of the numerical simulation method. The surface settlement curves of single-line and double-line shield construction had a "V" shape.
2. The surface settlement during shield construction is affected by many factors. The deformation modulus of the stratum had the largest influence on the surface settlement, and the tunnel’s burial depth affected the surface settlement and its rate of change. Increasing the tunnel’s burial depth can reduce the surface settlement in unfavorable stratum conditions.
3. The thrust force during shield tunneling should be maintained within an appropriate range. Increasing the shield thrust within the critical range can reduce surface settlement, but the surface uplift in front of the shield tunneling machine occurs when the critical value is exceeded. The higher the grouting quality, the larger the deformation modulus of the grouting layer and the lower the surface settlement. The proposed numerical simulation method is suitable for optimizing the shield construction parameters.
4. The numerical simulation results showed that a change in the cross-sectional spacing between the left and right lines did not significantly influence the surface settlement. More monitoring data on dual-line asynchronous shield construction should be collected to obtain further insights into the influence of cross-sectional spacing during excavation.

## Supplementary Information

Supplementary Information.

## Data Availability

The data that supports the findings of this study are available in the supplementary material of this article. They are true and available, and are authorized by all the authors (Supplementary material).
